# Crystal polymorphism and spectroscopical properties of sulfonamides in solid state by means of First Principles calculations

**DOI:** 10.1007/s10822-022-00465-2

**Published:** 2022-07-26

**Authors:** C. Ignacio Sainz-Díaz, Alexander Pérez de la Luz, Carolina Barrientos-Salcedo, Misaela Francisco-Márquez, Catalina Soriano-Correa

**Affiliations:** 1 Instituto Andaluz de Ciencias de la Tierra, Consejo Superior de Investigaciones Científicas-Universidad de Granada, Av. de las Palmeras, 4, 18100-Armilla, Granada, Spain; 2 Departamento de Química, Universidad Autónoma Metropolitana-Iztapalapa, Av. San Rafael Atlixco 186, Col. Vicentina, Ciudad de Mexico, 09340 México; 3 Laboratorio de Química Médica y Quimiogenómica, Universidad Veracruzana, C.P. 91700 Veracruz, Mexico; 4 Instituto Politécnico Nacional-UPIICSA, Té 950, Col. Granjas México, C.P. 08400 Mexico City, Mexico; 5 Unidad de Química Computacional, Facultad de Estudios Superiores Zaragoza, Universidad Nacional Autónoma de México, Iztapalapa, C.P. 09230 Mexico City, Mexico

**Keywords:** Sulfonamides, polymorphism, DFT calculations, infrared spectroscopy, hydrogen bonds

## Abstract

Sulfonamides are an important class of therapeutic agents. The increase in the number of new sulfonamide derivatives makes it necessary to study more rationally the chemical structure, because the solid forms often display different mechanical, thermal and physicochemical properties that can influence the bioavailability and stability of the drugs; consequently, the polymorphic structures are of great interest to the pharmaceutical industry because of their ability to modify the physical properties of the active pharmaceutical ingredient. The molecular interactions of these drugs in their crystal lattice are important for the stability of the crystals and polymorphism and for preparing composite complexes for optimizing the use of these drugs. In this work, the crystal structure of these drugs and crystal polymorphism is investigated. So, the crystal forms of antibiotics derivatives of the sulfonamides, sulfamethoxazole, sulfamethazine, sulfachloropyridazine, and sulfacetamide are studied at the molecular and supramolecular level by using computational modeling approach at quantum mechanical level. The spectroscopic properties of these systems are also studied explaining assignments of previous experimental data. The results of DFT calculations reproduce the crystal structures of sulfonamides determined experimentally and the polymorphism in these molecules have been clarified. Likewise, the main intermolecular interactions in all crystal forms of these sulfonamides are H-bonds among the sulfonic and amino groups and SNH groups, and also some π-π interactions. Also, these 3-D periodical models allow the exploration of the intermolecular interactions included in the crystal structures and some of these interactions can alter the vibration modes of the molecules. Therefore, the use of these models can be useful for experimental spectroscopy studies where use actual crystal solids.

**Supplementary Information:**

The online version contains supplementary material available at 10.1007/s10822-022-00465-2.

## Introduction

The polymorphism of the pharmacological compounds is one of the most important problems in the pharmaceutical industry for the design and development of new drugs. Organic molecules can pack in different crystal forms. These different packings are controlled by intermolecular interactions and the probability of their formation can depend on their relative energy and kinetics in the nucleation and crystal growth under a specific set of crystallization conditions. Then, the same molecule can form different crystal structures or polymorphs. This phenomenon of polymorphism is very interesting in pharmaceutical sciences, because each crystal packing can have different solubility, bioavailability, mechanical and rheological properties [1, 2]. The polymorphism of a drug substance or excipient can have a deep impact on its physical and physicochemical properties such as chemical hardness, density, melting point, adsorption, chemical reactivity, solubility, dissolution rate, biological action, production and formulation [3]. Likewise, a deficient knowledge of the change between the polymorphic forms of a drug may affect the pharmaceutical processing, the stability of the drug product, the bioavailability and the toxicity, and thereby the therapeutic efficacy of the bioactive substance [4]. Moreover, although polymorphic forms of a compound dissolve to give identical solutions, these solid forms differ concerning their thermodynamic stability, equilibrium solubilities, and rates of dissolution. Therefore, the release rate of a drug from a solid dosage form, whether in vivo or in vitro, can depend on its crystal polymorph. Hence, the control of crystal polymorphs is important for the chemical and pharmaceutical industry. On the other hand, within this searching rush of polymorphism, many authors have aimed to new polymorphs but sometimes can be pseudopolymorphs, or the same of other previously reported with different nomenclature, without a systematic solid characterization [5–7]. This polymorphism exists also in sulfonamides due to the presence of donor and acceptor atoms for intermolecular interactions in the packing motifs.

The polymorphism of sulfonamides received wide attention in the early 1940s, when sulfonamide chemotherapy was at its highest level. The sulfonamides represent an important class of medicinal compounds, which are extensively used as antibacterial agents. Likewise, millions of tons of sulfonamides are using and were used worldwide as effective antibiotics for treating veterinary diseases, and promoting growth in cattle, poultry, and swine [8]. The bacteriostatic activity of the arylsulfonamides is their biomimetism with the p-aminobenzoic acid in the folic acid metabolism path. In addition to their antimicrobial use, the sulfonamides have also many therapeutic applications as antifungal, antidiabetic, antiglaucoma, antiallergic, antiobesity, vasodilator, anti-HIV, and anticancer [9, 10]. In previous works we showed that the substitution of groups in the SO_2_N1H moiety for one of the H atoms on N1 may influence the tendency of the second H atom to participate in hydrogen bonding, that is, this region is an important interaction site. Also, the influence of the substituent group may be steric or electronic, as well as the bulky functional groups might be expected to hinder the approach of an amide H atom to the sulfonamide O atom of a neighboring molecule [11]. However, the functional group may, through the inductive effect or resonance effects at the molecular level, increase or reduce the electron density on the amide nitrogen, and consequently affecting the strength of the hydrogen bonds that might form. The combination of steric and electrostatic interactions and inductive or resonance effects may be sufficient in some substituted sulfonamides to preclude hydrogen-bond formation involving the amide hydrogen.

The discovery of new crystal forms of sulfonamides was, in most cases, completely fortuitous and, for many years, there was no major attempt to study the polymorphism of these compounds. Likewise, sulfonamides form co-crystals and salts of sulfa drugs that frequently display multiple related physicochemical characteristics including polymorphism and isostructural crystals, these properties supporting past and current interest in the solid-state chemistry of these molecules. In the last decades, many N4- and N1-substituted sulfonamides have been widely investigated due to their bacteriostatic activity against human and veterinary pathogens [12, 13]. Previous studies of crystal polymorphism in sulfonamides have been reported in experimental investigations [9, 14–16] and theoretical approaches [9, 17].

Molecular calculations have been applied to the study of the molecular [5] and crystal structures [17–19] of drugs describing the intramolecular and intermolecular interactions responsible for the stability of crystal polymorphs [20]. These calculations can describe also the atomic structures of clay minerals and their crystallographic properties [21] and the interactions of pharmaceutical drugs with the surfaces of phyllosilicates [22].

**Fig. 1:**
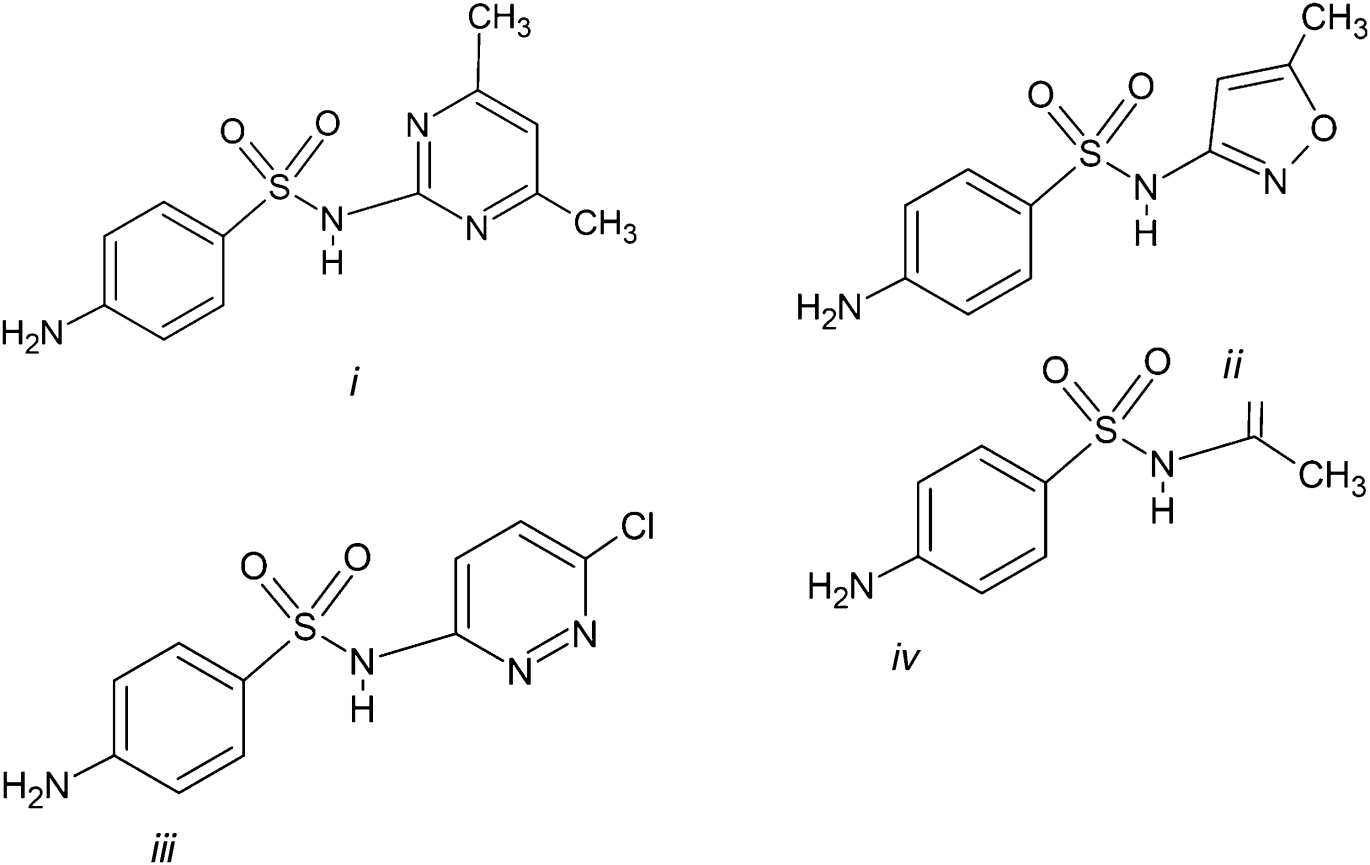
Molecular structures of (i) Sulfamethazine (SMT), (ii) Sulfamethoxazole (SMX), (iii) sulfachloropyridazine (SCP), and (iv) sulfacetamide (SCM)

The great importance of sulfonamides as antibiotics enhanced the research on crystal polymorphism of these drugs in the last decades [23]. However, some authors claimed new crystal forms, which were not really new polymorphs. Then, there are some discrepancies concerning the number of polymorphs in some sulfonamides. In this work, we have studied the crystal structures and intermolecular interactions of some representative sulfonamides, such as, sulfamethoxazole (4-Amino-N-(5-methyl-3-isoxazolyl)-benzenesulfonamide) (SMX), sulfamethazine (4-Amino-N-(4,6-dimethyl-2-pyrimidinyl)-benzenesulfonamide) (SMT), sulfachloropyridazine (4-amino-N-(6-chloro-3-pyridazinyl)-benzenesulfonamide) (SCP), and sulfacetamide (p-aminobenzene sulfonacetamide) (SCM) (Fig. 1), by using computational modeling methods. This selection set of sulfonamides tries to cover different OSN1 substitutions with heterocycles of different sizes and polarity, and linear amide groups. This selection can explore the effect of the substitutions on the intermolecular interactions where the SO_2_N1H group is involved. This study helps us to obtain a better understanding of the phenomenon of polymorphism of these crystal forms of antibiotics derivatives of sulfonamides and contribute to the decrease of infectious diseases because these sulfonamides are of great clinical importance.

## Models

Crystal structures of SMT [24–26], SMX [9, 15, 16, 27, 28], SCP [14], and SCM [29] were taken from previous experimental crystallographic X-ray diffraction data. The corresponding sulfonamides molecules were extracted from their crystal structures. Several works were reported as new crystal forms of these drugs. Then, these crystal structures were explored and compared applying periodical boundary conditions.

## Methodology

The crystal structures were calculated with Density Functional Theory (DFT) applying periodical boundary conditions based on plane wave conditions by using the Quantum-Espresso (QE) code version 6.4 [30] with the generalized gradient approximation (GGA), and the Perdew-Burke-Ernzerhof functional (PBE) for the exchange–correlation potential [31]. Plane wave PAW (Projector Augmented Wave) [32] pseudopotentials were used and dispersion corrections were included according to the DFT-D3 [33] scheme. In addition, different values of energy cutoff E_cut_(wfc) (40–120 Ry) and charge density (rho) cutoff (160–840 Ry) were tested to find the optimal calculation conditions (See Fig. S1 in Supporting Information). This preliminary study allowed optimization of the calculation parameters in order to obtain the maximum precision and reliability of the simulation with the minimum computational cost. The energy cutoff E_cut_(wfc) used was 100 Ry with a rho cutoff of 400 Ry, where the energy is independent of the cutoff values. Initially, preliminary calculations were performed at the Γ point of the Brillouin zone; however, some optimizations were not reproducible. Then, several *k* point grid samplings in the systematic optimization of the Brillouin zone were explored, finding the optimal calculations conditions (see Table S1 in Supporting information) and establishing the 3 × 1 × 1 and 1 × 3 × 1 *k* points as the best compromise between accuracy and computational effort.

The normal modes of vibration of the crystals were obtained from calculations based on the theory of density functional perturbation (DFPT) [34]. The powder X-ray diffraction patterns were simulated from the crystal structures by using the REFLEX code [35] considering a Copper radiation λ = 1.5406 Å.

## Results and discussion

### Sulfonamides crystal structures and polymorphism

The molecular structures of these antibiotics were studied previously as isolated molecules by means of computational chemistry methods, exploring conformational analysis, tautomerism and spectroscopic properties [36]. However, most of the infrared and Raman spectroscopic analyses are performed experimentally at solid state, mainly at crystalline state. Hence, our studies should be performed with the crystal structures of these compounds.

### Sulfamethazine

**Fig. 2:**
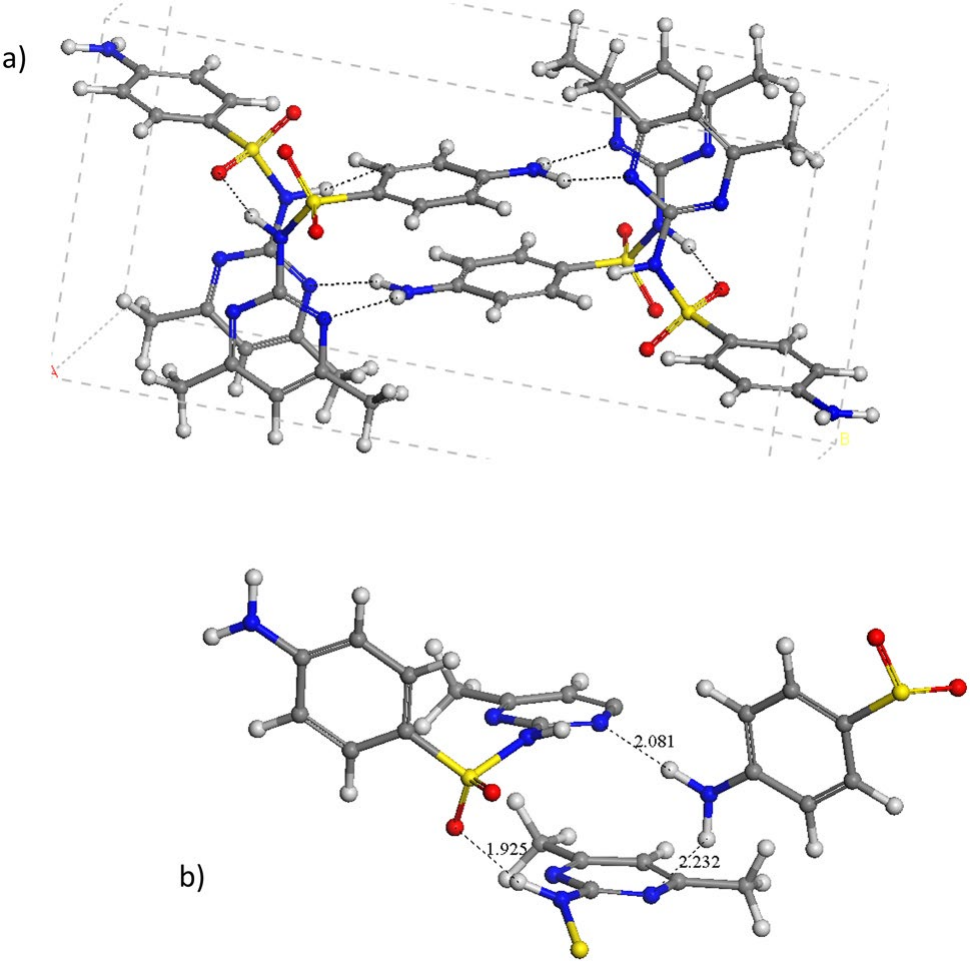
Optimized crystal form of sulfamethazine (**a**), indicating some intermolecular interaction motifs (**b**) (distances in Å). The C, S, N, Cl, and H atoms are represented in grey, yellow, blue, green, red, and white colours. This format is extended to the rest of the figures of this work.

In the case of sulfamethazine (SMT), three crystal forms have been claimed based on X-ray diffraction, CCDC num.1260687, code: SLFNMD01 [24]; CCDC num. 126,088, code: SLFNMD02 [26]; and CCDC num. 126,089, code: SLFNMD10 [25, 37] claimed two polymorphs I and II based on IR spectroscopy. The form II was obtained from form I by trituration. However, their IR spectra, thermograms and X-ray diffractograms were too similar and the distinction was not clear [23]. Nevertheless, Kuhnert-Brandstatter et al. [38] reported four forms of sulfamethazine by thermomicroscopic methods without solid characterizations. However, Maury et al. [26] reported the existence of only one polymorph and different crystal habits. Our previous work clarified these controversies finding that all these claimed crystal polymorphs are actually the same crystal form [36], and hence only one polymorph should be considered. Nevertheless, our previous calculations were performed at the Γ point of the irreducible Brillouin zone of the crystal structure. Exploring with more detail the Brillouin zone sampling with several *k* points grids along the crystallographic axes, we found that the energy of the crystal structure is 5.325 kcal/mol lower with 1 × 1×3 *k* points grid than with the Γ point (Table S1). Hence, we reoptimized the crystal structure of the SMT with 1 × 1× 3 *k* points matching closer to the experimental cell parameters than the previous one calculated with only the Γ point (Table 1 and Fig. 2). Nevertheless, the differences are small and the packing energy is similar in both calculations. In this work, we extend this study to the rest of the sulfonamides series in a similar way.

**Table 1:** Main cell parameters and packing energy of optimized and experimental crystal structures of SMT (distances in Å and angles in degrees) The footnotes have disappeared: a Polymorph references. b Number of molecules per unit cell. c Packing energy per molecule in kcal/mol (energy difference between the crystal unit cell and Z isolated molecules). d From Basak et al. [24]

Polymorph^a^	*a*	*b*	*c*	*α*	*β*	*γ*	*Z*^b^	*E*^c^
Exp (1,260,687)^d^	7.427	18.986	9.323	90.0	99.1	90.0	4	
Calc. (Γ point)	7.455	18.793	9.261	90.0	99.7	90.0	4	− 41.38
Calc. ( 1 × 1× 3 *k* points)	7.359	18.868	9.266	90.0	99.1	90.0	4	− 40.99

The optimized crystal structures show a similar XRD pattern that the experimental one with the same 2θ values and relative intensities (Fig. S2). It is remarkable, that in SMT the amino groups of aminobencenic rings do not form hydrogen bonds with sulfonic O atoms in contrast with the rest of the sulfonamides and polymorphs studied (see below). The heterocycle rings are parallel. The aminobencenic rings are parallel to each other being perpendicular with respect to the heterocyclic rings (Fig. 2). Each amino group joins 3 molecules, where each amino H atom forms hydrogen bonding with the heterocyclic N atoms of different molecules d(NH…N) = 2.081–2.232 Å (Fig. 2b). Only the sulfonamidic NH groups form hydrogen bonds with the sulfonic O atoms d(SO…HN) = 1.925 Å. According to the hydrogen bond topology [39], this hydrogen bond between the amido H atom and sulfonic O atom forms C(8) chains and those between the amino H atoms and the heterocyclic N atoms form chains C(10) and rings R^2^_2_(20) patterns.

### Sulfamethoxazole

Nine crystal polymorphs of sulfamethoxazole have been claimed previously based on X-ray diffraction reporting 8 crystal structures: CCDC num. 1,260,679, CSD_code: SLFNMB01; CCDC num. 1,260,680, CSD_code: SLFNMB02; CCDC num. 1,260,681, CSD_code: SLFNMB03; CCDC num. 1,260,682, CSD_code: SLFNMB04 [28]; CCDC num. 270,106, CSD_code: smaIII [16]; CCDC num. 270,107, CSD_code: smaIV [16]; CCDC num. 930,472, CSD_code: BnzSO2NOCH3a [15]; CCDC num. 978,497, CSD_code: datos_0m [9]. Recently a new structure of this polymorphic antibiotic sulfamethoxazole has been claimed [27] (CCDC num. 1,979,417, CSD code SLFNMB09). Three of these polymorphs (I, II, and III) were identified previously based on thermoanalysis and spectroscopy studies [23]. Besides, there is no agreement related with the nomenclature of these polymorphs. Therefore, a preliminary exploration of all these polymorphs claimed is performed comparing all these crystal forms in this work.

Initially, preliminary calculations were performed at the Γ point of the irreducible Brillouin zone of the crystal structure. However, the optimization of some polymorphs gave structures far from experimental ones, except the structures belonging to forms II and III that matched the experimental cell parameters. Exploring with more detail the Brillouin zone sampling with several *k* points grids along the crystallographic axes we found that the energy of the crystal structures belonging to form I was -61.761 kcal/mol lower with 1 × 3 × 1 *k* points grid than with the Γ point (Table S1). Analogously, the energy of the polymorph IV was 34.182 kcal/mol lower with 3 × 1 × 1 *k* points than with the Γ point (Table S1). A higher number of *k* points did not yield lower energy and demanded higher computational effort. Hence, we reoptimized the crystal structures of the polymorphs I with 1 × 1 ×  3 *k* points and the form IV with 3 × 1 × 1 *k* points.

**Fig. 3:**
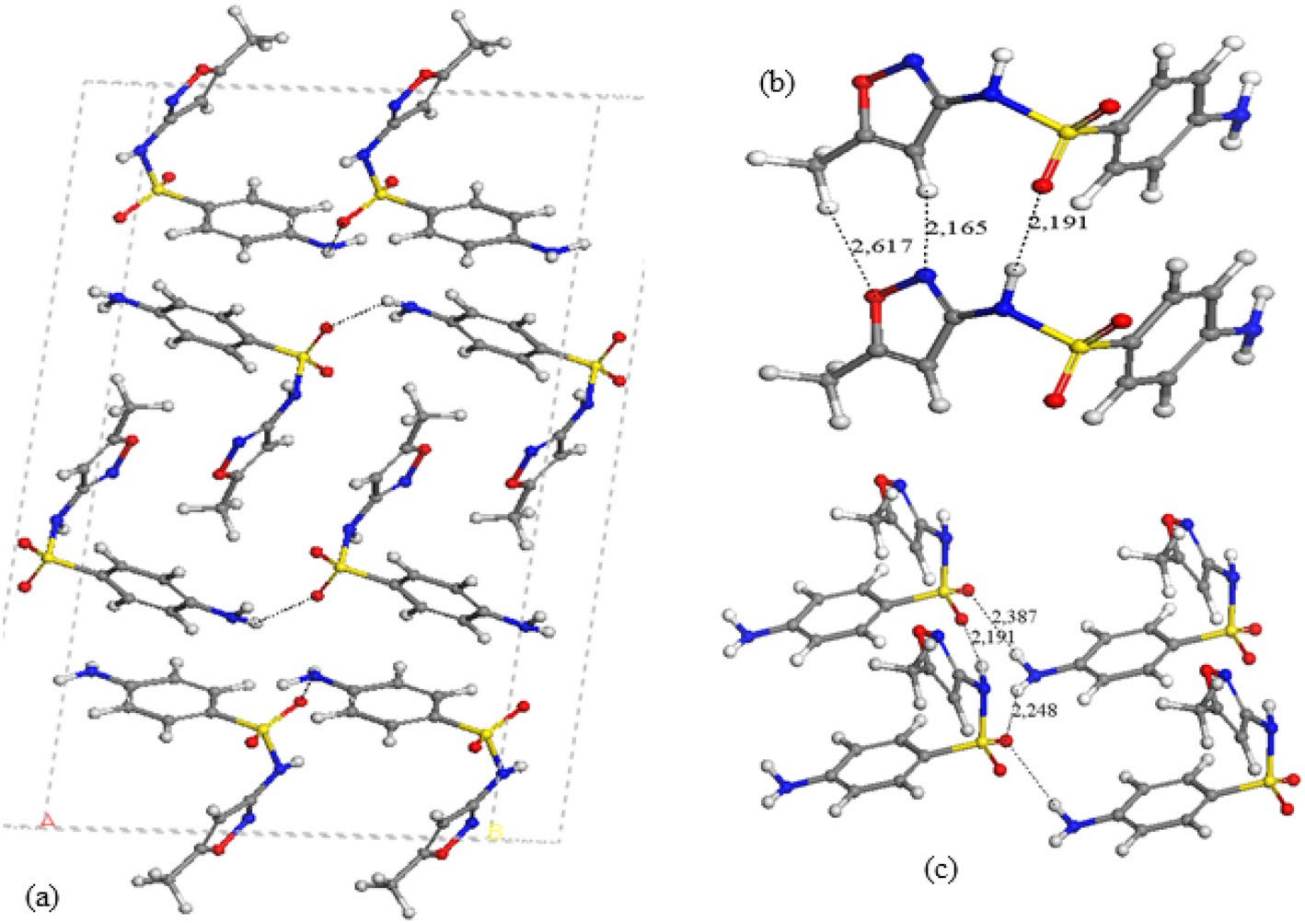
Optimized crystal structure of SMX polymorph I (**a)** indicating some intermolecular interaction motifs (**b** and **c**)

All these polymorphs were fully optimized including atomic positions and cell parameters reproducing the experimental data (Table 2). In Fig. 3, these optimized structures are described. Our calculations reproduced the experimental crystal structures in all polymorphs. Actually, only 4 crystal forms should be considered as different polymorphs, instead of the initial 9 forms previously claimed. The crystal forms, 1,260,679, 1,260,681, 930,472, and 1,979,417, have very similar cell parameters, and the same space group, C2/c, belonging to the same type of polymorph, previously named as Form I. The cell parameters of the crystal ‘1,979,417’ are slightly smaller than the rest because it was measured at 100 K whereas the rest were measured at 295 K. Analogously, the crystal forms, 1,260,680, 1,260,682, and 978,497, have also similar cell parameters, the same space group, C2/c, and can be considered as the same polymorph, previously named as Form II. The crystal ‘978,497’ shows smaller cell parameters because was measured at 100 K being the rest analysed at 295 K. The other polymorphs are ‘270,106’ (Form III) and ‘270,107’ (form IV). The Form II has the highest packing energy. This behaviour is consistent with previous experimental results, where the Form II was more stable than Form I with a transition energy of ~ 1 kcal/mol [23, 39]. The packing energy follows the sequence: form II > III > IV > I (Table 2). Nevertheless, the energy differences are not important and hence, the formation of one polymorph of SMX will depend more on experimental conditions than the thermodynamic control.

**Table 2:** Main cell parameters of optimized and experimental (in brackets) crystal structures of polymorphs of SMX (distances in Å and angles in degrees)

Polymorph^*a*^	*a*	*b*	*c*	*α*	*β*	*γ*	*Z*^*b*^	*E*^*c*^
SLFNMB01 (1,260,679) form I	15.99 (16.06)	5.47 (5.48)	25.81 (25.76)	90.0 (90.0)	95.4 (96.1)	90.0 (90.0)	8	− 37.90
SLFNMB03 (1,260,681) form I	15.98 (16.05)	5.48 (5.47)	25.82 (25.75)	90.0 (90.0)	95.2 (96.1)	90.0 (90.0)	8	− 37.88
BnzSO2NOCH3a (930,472) form I	16.01 (16.08)	5.47 (5.48)	25,78 (25.76)	90.0 (90.0)	95.5 (96.1)	90.0 (90.0)	8	− 37.89
SLFNMB09 (1,979,417) form I	16.01 (15.90)	5.47 (5.46)	25.64 (25.33)	90.0 (90.0)	95.90 (96.4)	90.0 (90.0)	8	− 37.87
SLFNMB02 (1,260,680) form II	24.81 (25.09)	7.29 (7.23)	14.76 (14.85)	90.0 (90.0)	118.1 (118.0)	90.0 (90.0)	8	− 39.03
SLFNMB04 (1,260,682) form II	24.85 (25.11)	7.29 (7.23)	14.74 (14.85)	90.0 (90.0)	118.1 (117.9)	90.0 (90.0)	8	− 39.03
datos_0m (978,497) form II	24.86 (24.72)	7.28 (7.20)	14.72 (14.66)	90.0 (90.0)	118.2 (118.2)	90.0 (90.0)	8	− 39.11
smaIII (form III) (270,106)	11.62 (11.64)	6.94 (6.82)	15.18 (15.42)	90.0 (90.0)	107.3 (107.1)	90.0 (90.0)	4	− 38.88
smaIV (form IV) (270,107)	5.50 (5.49)	16.87 (16.76)	12.54 (12.42)	90.0 (90.0)	97.4 (97.1)	90.0 (90.0)	4	− 38.07

^a^Polymorph references

^b^Number of molecules per unit cell

^c^Cohesive energy per molecule in kcal/mol (energy difference between the crystal unit cell and Z isolated molecules)

The powder X-ray diffraction patterns of these optimized SMX polymorphs were simulated and compared with the experimental ones (Fig. S3). The optimized crystal structures show similar XRD patterns to the experimental structures with the same 2θ values of the reflections and only changing the relative intensities of the peaks. The four crystal structures of the polymorph I show a similar XRD pattern with only differences in the relative intensities of the peaks confirming that all belong to the same polymorph. Analogously, the three optimized crystal forms of polymorph II show similar XRD patterns to the experimental structures and all these show similar XRD patterns each other, indicating that they belong to the same polymorph II. On the contrary, the forms I, II, III, and IV show different XRD patterns corresponding to different crystal polymorphs.

In all polymorphs of SMX, the molecule has a syn conformation, where the N–H group is oriented to the same side as the heterocyclic N atom. This is consistent with our previous calculations, where the syn conformer was the most stable one [36]. The crystal structure of form I shows the heterocyclic rings alternating in opposite orientations and bridging the amino-benzenic rings (Fig. 3a). A motif with three hydrogen bonds is observed between the heterocyclic moieties: one between the sulfonic O atom and the H atom of the NH group d(SO…HN) = 2.191 Å forming a chain C(4) pattern, one between the heterocyclic N atom and the heterocyclic CH H atom d(N…HC) = 2.165 Å, and another one between the heterocyclic O atom and the methyl H atom d(NO…HCH_2_) = 2.617 Å (Fig. 3b). These hydrogen bonds form a ring pattern R^2^_2_(7) of 7 atoms attached to another ring R^2^_2_(10) of 10 atoms (Fig. 3a). The sulfonic O atoms also forms hydrogen bonds with the amino H atoms forming a chain C(8) motif SO…HNH…O…HNH…OS, d(SO…HN) = 2.248, 2.387 Å, and at the same time form a ring R^3^_3_(10) pattern (Fig. 3c).

**Fig. 4:**
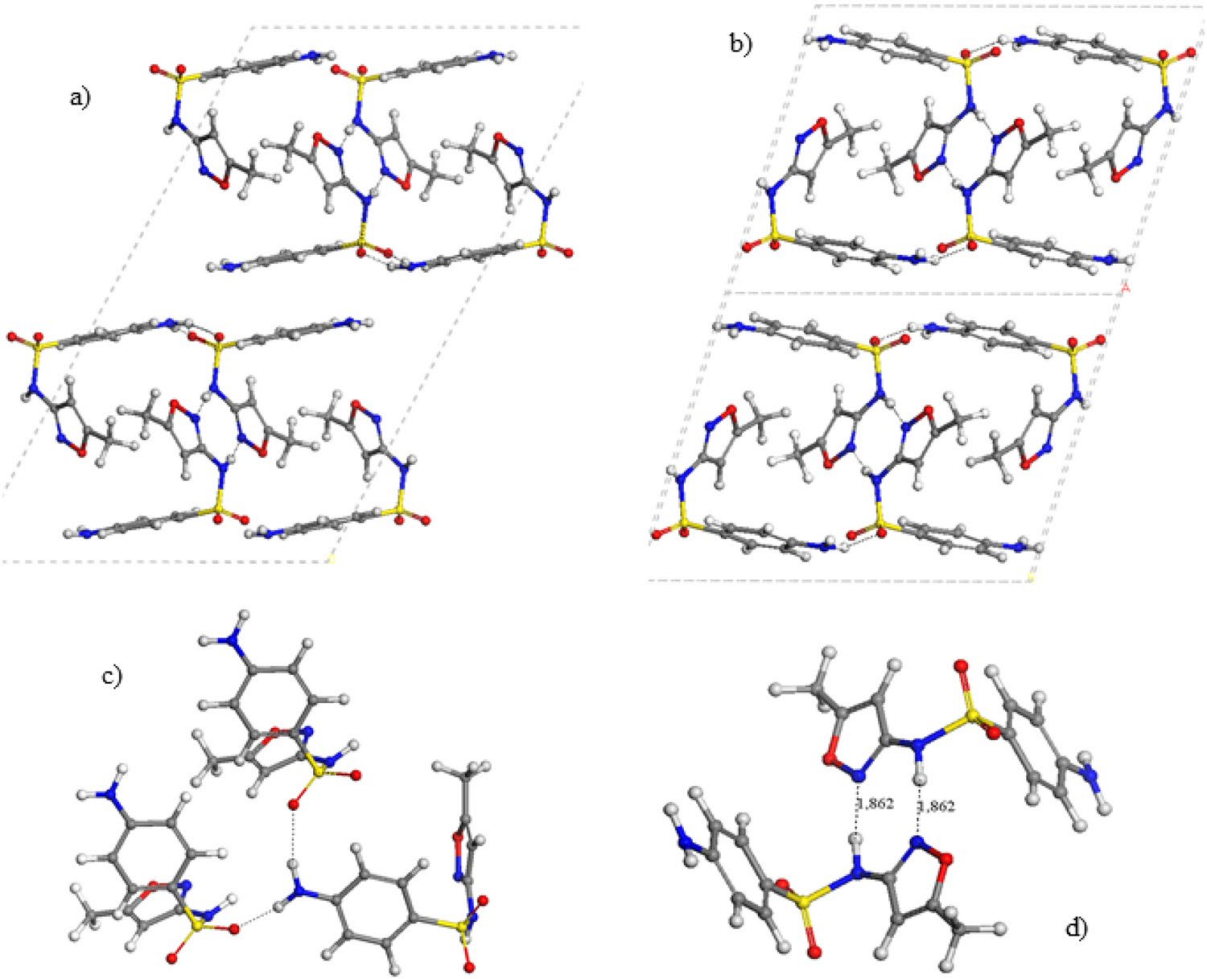
Optimized crystal structure of SMX polymorphs II (**a**) and III (**b**), highlighting some intermolecular motifs (**c** and **d**)

In the polymorphs II and III the relative orientations of the heterocyclic and aminobenzenic rings are similar with different packing. The heterocyclic rings are parallel each other in alternating orientations bridging the aminobenzenic rings, which are also parallel each other. Layered packing can be observed where the aminobenzenic rings of one layer interact with the homologue ring of the other layer by electrostatic forces between the amine and sulfonic groups (Figs. 4a and b). In both polymorphs, the intermolecular interaction motifs are different that in form I. The sulfonic O atoms forms hydrogen bonds with the amine H atoms forming a C(8) chain OSO…HNH…OSO…HNH…, d(SO…HN) = 1.961, 2.009, 2.103 Å (Fig. 4c). The heterocyclic rings form a R^2^_2_(8) ring motif with two hydrogen bonds, between the NH H atom and the N atom, d(N…HN) = 1.860, 1.862 Å, that can be considered as pseudotautomeric forms (Fig. 4d). Our previous studies on tautomers of SMX found that the sulfonamide tautomer was 7.07 kcal/mol more stable than the sulfonimide tautomer [36]. However, the cohesive energy for a pair of SMX molecules is -78 kcal/mol (Table 2), being higher than the energy barrier (53 kcal/mol) for the intramolecular transition between both tautomers [36]. This indicates that H atom exchanges can occur between both N atoms for each pair of molecules. Although the exact position of the H atom is difficult to be determined with XRD, this tautomeric consideration is corroborated by some bond lengths, where the SN–C bond length is shorter and the C-NO bond of the heterocycle is longer in the polymorphs II and III than in the form I, indicating a certain tautomeric participation (Table 3). In general, the hydrogen bonds in form II are shorter than in form I justifying the higher packing energy of form II (Table 2) being consistent with the IR spectroscopy study of Yang and Guillory [23].

**Table 3:** Main geometrical features (distances in Å and angles in º) of the molecules in the crystal structures of sulfamethoxazole optimized

	SMX-I	SMX-II	SMX-III	SMX-IV	SMX exp^c^
S-O_1_	1.467	1.461	1.460	1.463	1.441
S-O_2_	1.459	1.460	1.461	1.462	1.435
S-C	1.755	1.747	1.747	1.745	1.747
S–N	1.687	1.689	1.686	1.677	1.651
N–C	1.402	1.389	1.387	1.392	1.407
NC-N_het_	1.326	1.331	1.332	1.329	1.307
N–O	1.413	1.413	1.412	1.417	1.414
O-C	1.364	1.360	1.359	1.361	1.356
N–H…OS	2.191 (2.248, 2.387)^a^	(1.961, 2.103)^a^	2.009^a^	2.134 (1.987, 2.405)^a^	2.48
NH…N	2.165^b^	1.862	1.860		
CON…HC	2.658, 2.617	2.805		2.204	
CH…C_arom_	2.727, 2.837	2.896, 2.957			
O-S–O	119.4	119.9	120.0	119.7	119.4
S–N-H	111.8	112.7	114.0	115.2	113.2
C-S–N	107.8	107.2	106.9	108.2	107.1
C-S–N-C	55.9	60.4	60.9	63.0	55.0
C–C-S–N	101.0	98.1	106.5	104.9	105.2
S–N-C-N_het_	139.4	148.0	149.8	159.7	140.7
H-N–C-N	3.1	6.7	3.8	3.0	2.1
H_2_N-S-C_het_^*d*^	84.7	84.8	82.8	88.4	81.0
C_α_-C’_α_-N-X^*e*^	23.1	20.2	14.8	13.3	8.7

^a^NH_2_ group

^b^ON…HC

^c^Experimental data from Das et al. [9]

^d^Angle between the central axes of both rings

^e^Coplanarity of both rings, both aromatic C atoms in alpha position with respect to the sulfoxide group and the heterocyclic N and C atoms in SMX.

**Fig. 5:**
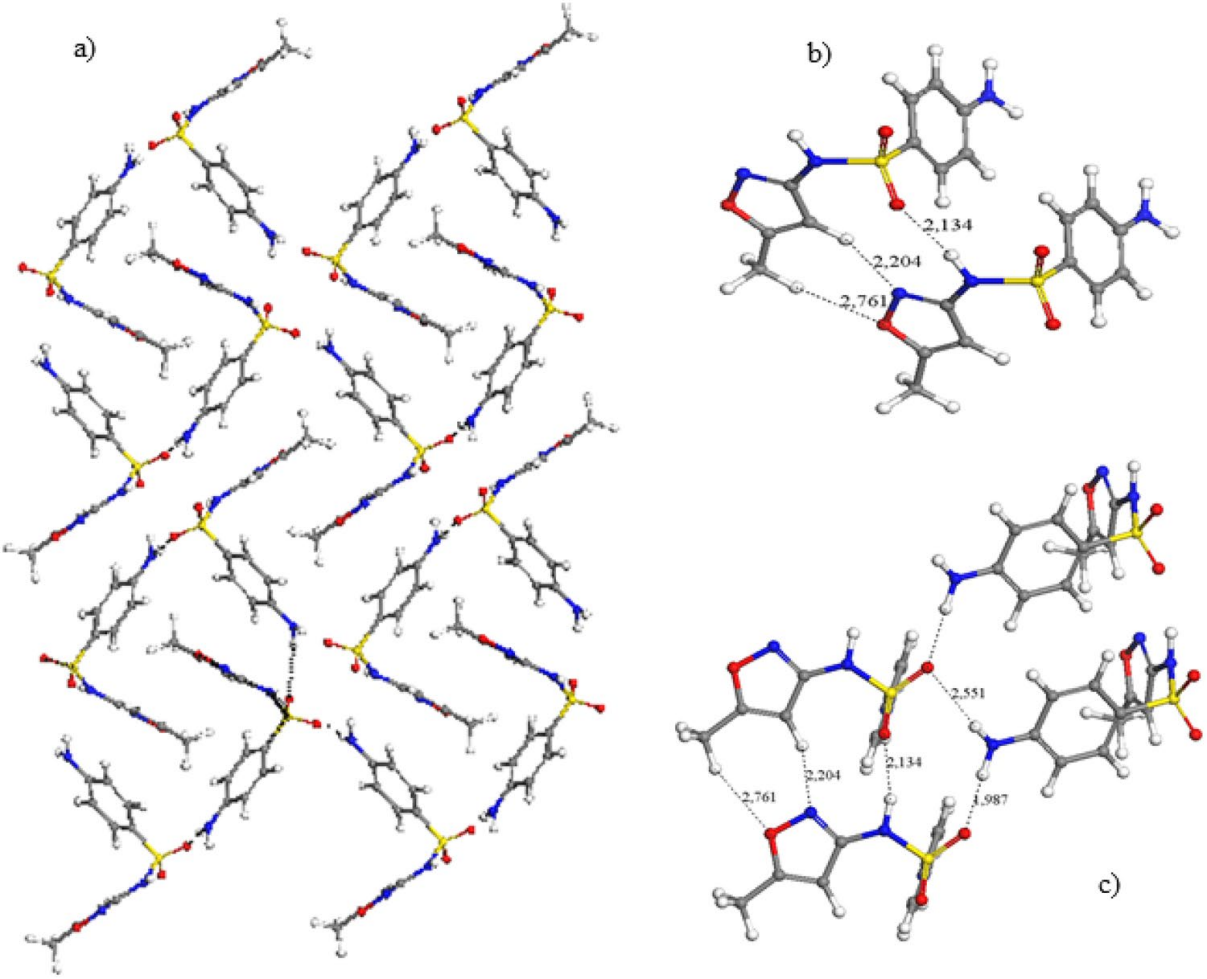
Optimized crystal structure of SMX polymorph IV (**a**) highlighting some intermolecular motifs (**b**, **c**)

On the other hand, the polymorph IV shows a helical configuration of the SMX molecules (Fig. 5a). The main intermolecular interaction motifs (Figs. 5b, c) are similar to those found in the polymorph I (Fig. 3b, c). The amine H atoms form a hydrogen bonds C(8) chain with the sulfonyl O atoms, N–H…O(S)…HNH…OS, d(SO…HN) = 1.987, 2.551 Å forming also a ring R^3^_3_(10) pattern. The heterocyclic rings form a motif with three H bonds d(SO…HN) = 2.134 Å, d(N…HC) = 2.204 Å, and d(NO…HCH_2_) = 2.761 Å, forming two ring patterns R^2^_2_(7) and R^2^_2_(10) as in form I.

The molecular structure of SMX is similar in all crystal polymorphs, only small differences can be detected (Table 3). In form I, the sulfonic groups are asymmetric with different S–O bond lengths, due to the different nature of the hydrogen bonds in which they participate, d(SO…HN) = 2.191 Å and d(SO…HNH) = 2.248, 2.387 Å. The bonds S-C and N–C are slightly longer in form I than in the rest of the polymorphs. The main intermolecular hydrogen bonds are shorter in form II than in form I (Table 3), corroborating previous experimental results specially where the NH group is involved [23, 39]. The conformations of the functional groups are similar with analogous dihedral angles in the SMX molecules of all polymorphs.

### Sulfachloropyridazine

In the case of SCP, two polymorphs were considered, which were claimed previously with experimental crystallographic studies: CCDC num. 274,449 Tan et al. [40], and SeethaLekshmi et al. [14] recently showed a crystal form CCDC num. 1,841,485 different that the previously reported. Following a similar procedure to above we explored the Brillouin zone sampling with several *k* points grids along the crystallographic axes directions finding the best optimization results for the polymorph I, using 3 × 1 × 1 *k* points grid as in the above form IV of SMX. In the case of the polymorph II, we found that the energy of the crystal structure was -104.296 kcal/mol lower with 1 × 3 × 1 *k* points grid than with the Γ point (Table S1).

The optimized crystal structures with our DFT calculations matches the experimental cell parameters with a standard deviation smaller than 1% (Table 4). The simulated XRD patterns of these optimized forms are similar to the simulated from the experimental crystal structures with the only variations in the relative intensities. The XRD patterns of the forms I and II of SCP are clearly different being actually different polymorphs (Fig. S4). The packing energy is similar for both polymorphs (Table 4) being lower than that of SMX crystals (Table 2). This is according with the experimental values of sublimation enthalpy of similar sulphonamides (32.3 kcal/mol) [15]. Nevertheless, the SCP form I is more stable than the SCP form II. This is consistent with experimental behaviour where the more stable is also the form I with a lower heat of fusion [14]. Nevertheless, the energy difference is small and additional kinetic and thermodynamic factors will involve at higher temperatures. The SCP molecules adopt the conformer syn where the heterocyclic N atoms are on the same side as the sulfonamide NH group. This is consistent with our previous calculations of isolated molecules where this syn conformer is more stable than anti [36].

**Table 4:** Main cell parameters of experimental and optimized crystal structures of polymorphs of SCP and SCM (distances in Å and angles in degrees)

Polymorph^*a*^	*a*	*b*	*c*	*α*	*β*	*γ*	*Z*^*b*^	*E*^*c*^
SCP (274,449)^d^ form I	5.55 (5.55)	17.31 (17.10)	12.66 (12.61)	90.0 (90.0)	92.3 (92.6)	90.0 (90.0)	4	− 32.32
SCP (1,841,485)^e^ form II	16.10 (16.10)	5.65 (5.60)	26.82 (26.70)	90.0 (90.0)	95.1 (95.7)	90.0 (90.0)	8	− 31.48
SCM (1,260,699)^f^	7.97 (7.93)	7.97 (7.93)	16.29 (16.45)	90.0 (90.0)	90.0 (90.0)	90.0 (90.0)	4	− 36.24

^a^Polymorph references

^b^Number of molecules per unit cell

^c^Packing energy per molecule in kcal/mol (energy difference between the crystal unit cell and Z isolated molecules)

^d^Experimental data from [40]

^e^Experimental data from [14]

^f^Experimental data [29]

**Fig. 6:**
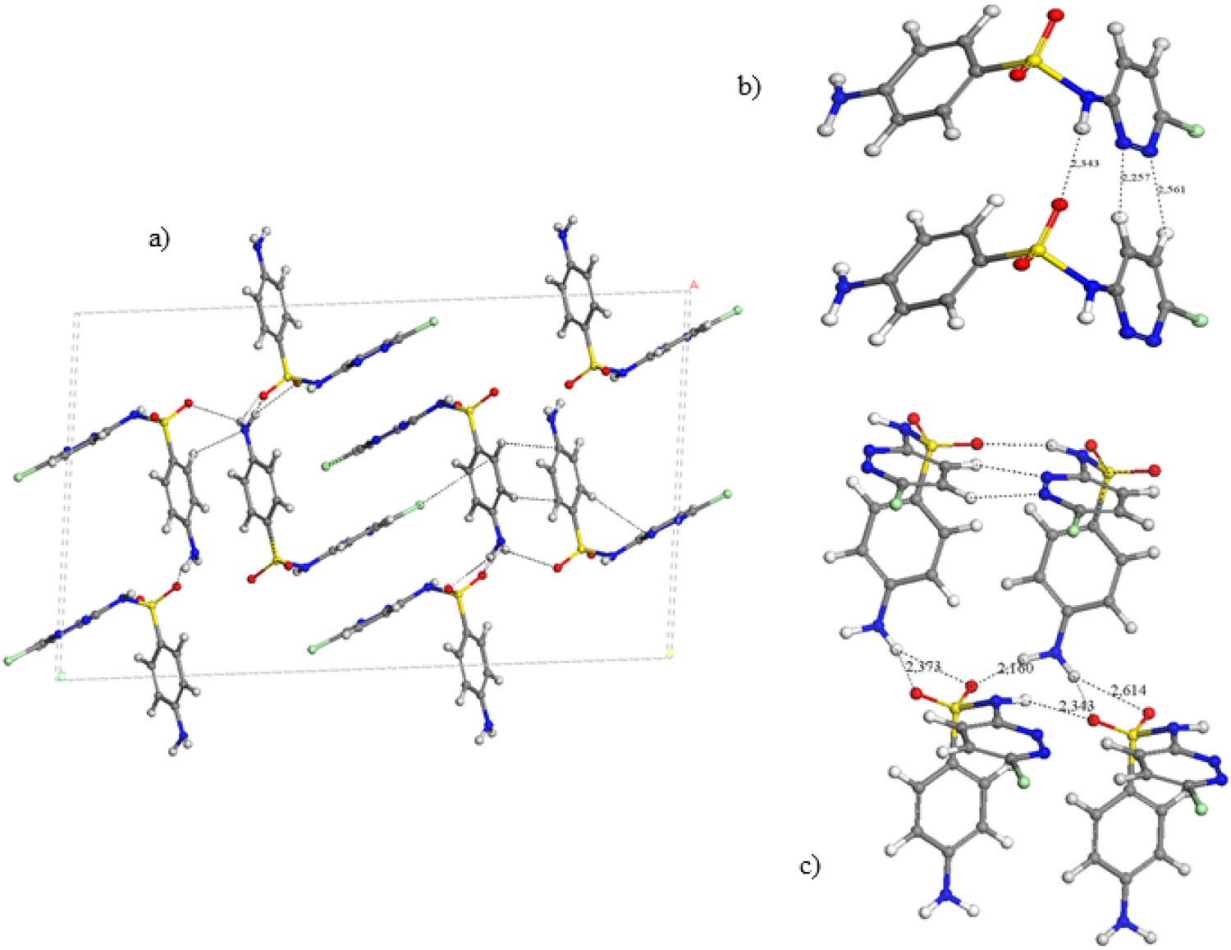
Optimized crystal structures of the polymorph I of SCP (**a**) highlighting some intermolecular motifs (**b**, **c**)

**Fig. 7:**
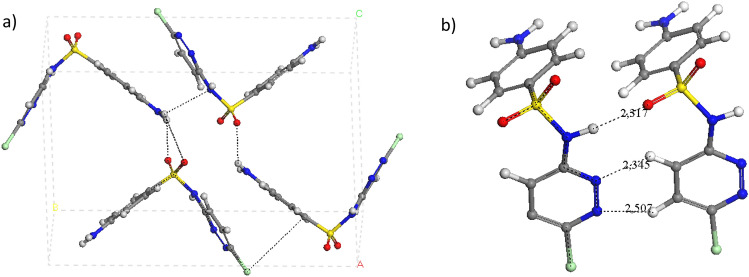
Optimized crystal structures of the form II of SCP-II (**a**), highlighting some intermolecular motifs (**b**)

In the polymorph SCP-I, the aminobenzenic rings are parallel each other in alternating orientation. In a similar way the heterocyclic rings are parallel each other. However, in the polymorph SCP-II, the aminobencenic and heterocyclic rings are parallel at a distance of 3.535 Å. In both polymorphs the heterocyclic rings are joined by a motif of three intermolecular hydrogen bonds, d(SO…HN) = 2.343 (form I), 2.317 (form II) Å and d(CH…N_het_) = 2.257–2.561 Å (form I), 2.345–2.507 Å (form II). Besides, the amino H atoms form intermolecular hydrogen bonds with the sulfonic O atoms in both polymorphs (Figs. 6 and 7) as in the above sulfonamides. The Cl atoms interact with the π electron clouds of both aromatic rings, the aminobencenic one and the heterocyclic one, with Cl…π interactions, d(Cl…π) = 3.800 Å (with heterocyclic), 3.480 Å (with aminobencenic ring) in form I, and d(Cl…π) = 3.570 Å (with heterocyclic), 3.449 Å (with aminobencenic ring) in form II. In general, the molecule structure and intermolecular interactions are very similar in both polymorphs and the only difference is in the packing of the crystal lattice. In both forms, the amidic H atom and sulfoxy O atom form a C(4) chain and also a ring R^2^_2_(10) pattern with the heterocyclic N atoms (Fig. 6b) which form attached a ring R^2^_2_(6) motif.

The powder X-ray diffraction patterns of the optimized SCP crystal structure were simulated and compared with the simulated from the experimental data (Fig. S4a, b). The optimized crystal structures show a similar XRD pattern that the experimental ones with the same 2θ values and relative intensities. This confirms that both crystal forms are different and both are actual polymorphs.

### Sulfacetamide

**Fig. 8:**
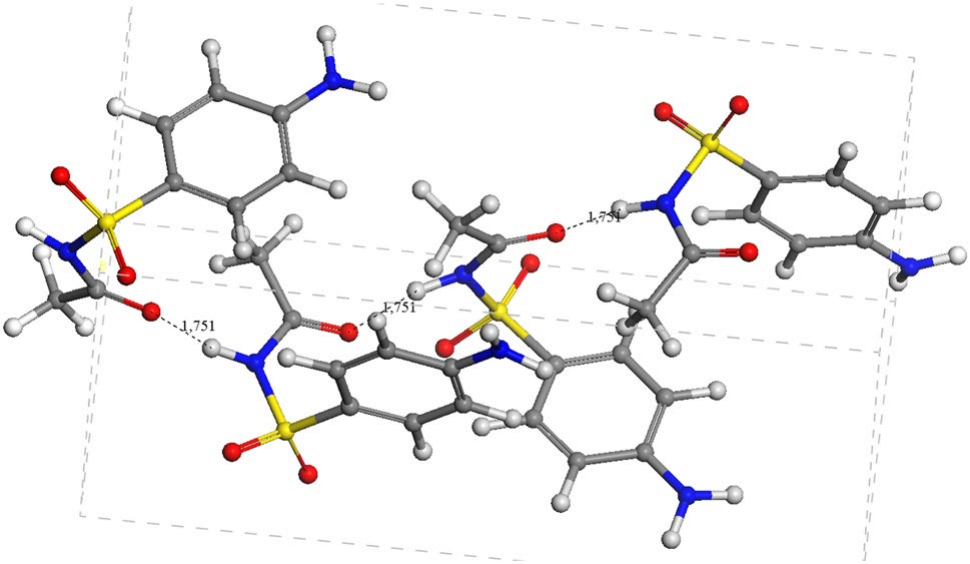
Optimized crystal structures of SCM

In the case of SCM, only one crystal form was found CCDC num: 1,260,699 (CSD code: SLFNMG01) [29]. The optimizations of this crystal structure using the Γ point of the Brillouin zone and using 3 × 1 × 1 *k* points yielded similar structures. The cell parameters of the calculated and optimized crystal structure reproduce the reported experimentally with a standard deviation smaller than 1% (Table 4). The cohesive energy is higher than in SCP but slightly lower than in SMX. In this crystal lattice (Fig. 8), the SCM molecules have a conformation anti with the carbonyl group on the opposite side with respect to the sulfonamide SN–H group. No energetic preference was found in our previous calculations of isolated molecules where both conformers, syn and anti, had the same energy [36]. In this crystal lattice the main intermolecular interactions are the strong hydrogen bonds between the carbonyl O atoms and the SNH H atoms with d(CO…HN) = 1.751 Å forming a C(4) chain, and the hydrogen bonds between the amine H atoms and the sulfonic O atoms forming a C(8) chains, as in the above sulfamides (Fig. 8). The N–C bond length is shorter than in other sulfonamides, due to the participation of the carbonyl π electrons in this bond. The powder X-ray diffraction pattern of the optimized SCM crystal structure was similar that the experimental one with the same 2θ values and relative intensities (Fig. S4c; Table 5).

**Table 5:** Main geometrical features (distances in Å and angles in º) of the molecules in the crystal structures of sulfonamides optimized

	SMT	SMT _exp_^a^	SCP-I	SCP_exp_^b^	SCP-II	SCM
S-O_1_	1.467	1.435	1.465	1.434	1.461	1.464
S-O_2_	1.463	1.431	1.459	1.433	1.460	1.459
S-C	1.750	1.746	1.743	1.734	1.750	1.744
S–N	1.678	1.632	1.687	1.647	1.695	1.696
N–C	1.396	1.406	1.392	1.394	1.405	1.376
NC-N_het_	1.341–1.346	1.324–1.333	1.344	1.324	1.342	
N–O			1.342^c^		1.339^c^	
O-C			1.737^d^		1.735	1.239^e^
N–H…OS	1.93	2.190	2.429, 2.932, 2.963	2.43	2.343, 2.373–2.614^f^	1.909, 2.212
NH…N	2.08, 2.23	2.37, 2.53				1.751^e^
CON…HC					2.257, 2.561	
CH…C_arom_					2.734, 2.951	
O-S–O	118.9	119.0	119.7	119.4	118.7	118.3
S–N-H	113.1	111.8	113.5	114.2	111.6	113.9
C-S–N	108.6	108.2	107.1	106.7	106.8	106.0
C-S–N-C	83.9	83.0	62.5	63.5	54.3	53.1
C–C-S–N	128.5	129.3	101.5	102.3	104.6	111.9
S–N-C-N_het_	36.2	34.9	156.1	85.0	140.6	10.9^e,g^
H-N–C-N	18.8	17.3	3.7	4.4	2.7	168.5^e,g^
H_2_N-S-C_het_^h^	101.2	102.0	85.8	83 (I), 74 (II)	81.1	95.3
C_α_-C’_α_-N-X^i^	48.0	47.3	8.2	16.4	4.8	43.8

^a^Extracted from the experimental structure from [29]

^b^Experimental data from [14]

^c^N–N bond length

^d^C–Cl bond length

^e^Carbonyl O atom instead of heterocyclic N atom

^f^With the amino NH_2_ group

^g^Conformer anti

^h^Angle between both rings

^i^Coplanarity of both rings, both aromatic C atoms in alpha position with respect to the sulfonyl group and the HN substituent

## Spectroscopical properties

In many theoretical works, experimental IR spectra are compared with calculated values from isolated molecules, however, most of the experimental data are performed from solid crystals, instead of isolated molecules in the gas phase or in highly diluted dissolutions. Hence in this work, frequencies of the main vibration modes of the solid crystal structures were calculated for these crystal polymorphs of these sulfonamides in Raman (Table S2) and IR spectroscopy (Tables S3 and S4) in a more realistic way.

We analysed the frequencies of SMT previously calculated using the Γ point of the Brillouin zone [36]. In this work we compare those frequencies with new calculations performed at a higher level using  1 × 1× 3 *k* points of the lattice Brillouin zone. In both cases similar values were obtained (Table S3). In general, the bands are combinations of vibrations for the same type of bond in the different molecules forming the crystal unit cell within a range of frequencies. Until now, only partial experimental assignments of some IR bands of SMT have been reported by Maury et al. [26]. Nevertheless, we extracted frequency values from the experimental spectrum of SMT in solid state with KBr [28] without previous assignment reported for a better comparison with the calculated values. This comparison has allowed the assignments of most of the experimental IR bands. Besides, we have included also the experimental frequency values of sulfadiazine, a similar structure without methyl groups for an additional comparison [41]. In the high frequency modes the *ν*(NH_2_)_*as*_ > *ν*(NH_2_)_*s*_ > *ν*(SN–H) frequency sequence is observed that is similar in all sulfonamides studied. Previous experimental works have assigned the ν(CH) mode of the heterocyclic C-H bond between the bands assigned to ν(CH) of the aromatic C–H bonds, whereas our calculations indicate that *ν*(CH)het > *ν*(CH)arom. This fact is observed in all calculated sulfonamides. These bands were also observed experimentally however the assignments were the contrary, *ν*(CH)het < *ν*(CH)arom. Hence, our calculations clarify these miss-assignments. On the other hand, our calculations can distinguish the *ν*(CH) vibration mode of aromatic C-H bonds that are oriented as anti or syn with respect to the SN–H group, being higher the frequencies of the anti-position. In addition, the H atoms in the positions close to the sulfonyl group have a slightly higher *ν*(CH) frequency than those close to the amino group. These differences could not be assigned experimentally. Besides, another miss-assignment is found in the experimental IR studies, where the *ν*(CH) of methyl group was assigned to *ν*(CH) of aromatic bonds. Hence, our assignment is that the low frequency range of aromatic *ν*(CH) bands (3066–3030 cm^−1^) should be assigned to the ν(CH_3_)_*as*_ of methyl groups. At the same time, the experimental bands at 2920–2900 cm^−1^ should be assigned to the ν(CH_3_)_*s*_. On the other hand, the experimental frequency values of sulfadiazine followed the sequence δ(NH_2_)_*s*_ < *ν*(CC)arom < *ν*(CC)het, being the contrary to our calculated frequencies of SMT probably due to the presence of methyl groups in sulfadiazine. Analogously, the bands at 1262–1304 cm^−1^ assigned experimentally to the ν(CN) mode should be assigned to ν(SO)_*as*_, according to our calculations.

In SCM, *ν*(SN–H) appears at significantly lower frequency than others due to the participation of this group in stronger hydrogen bonds in the crystal packing of SCM (Table S3). The calculated frequencies are close to the experimental ones [43], and our calculations have helped to the assignments of the experimental bands. The *ν*(NH_2_) frequencies are higher in SCM than in SMT probably due to the stronger intermolecular hydrogen bonds through these groups in SMT than in SCM. The ν(C=O) mode calculated in the crystal structure of SCM integrates several combinations of the modes of the carbonyl groups of the molecules that form the crystal structure showing a range of 1669–1636 cm^−1^.

In SCP, taking into account that the intermolecular interactions are similar for both polymorphs (see above) we can consider that the frequencies of the main modes will be similar for both polymorphs. Hence, we have calculated only the IR frequencies of the polymorph I for comparison (Table S3). The calculated frequencies are close to the experimental values [14, 42]. The calculated frequencies higher that 3300 cm^−1^ are higher than the experimental ones. Some discrepancies have been found in the experimental values [14] assigned. We found that *ν*(NH_2_)_*s*_ > *ν*(SN–H), whereas Basha et al. [42] assigned these bands in the opposite sequence. Our calculations are consistent with the Seethalekshmi et al. [14] assignments. Both ν(NH_2_) modes (symmetric and antisymmetric) follow the sequence SMT < SCM < SCP. On the other hand, the ν(SN–H) mode follows the sequence SCM < SMT < SCP. Both sequences are related with the hydrogen bonding intermolecular interactions where these groups participate in the crystal lattice. In SMT the hydrogen bonds of amine H atoms with sulfonic O atoms are stronger than in SCP, hence the frequency of ν(NH_2_) will be lower in SMT. In isolated molecules with a lack of these intermolecular interactions, these frequencies are higher being for ν(NH_2_)_*as*_ 3624 cm^−1^ in SMT molecule [36], and 3498–3496 cm^−1^ in SMT crystal. In the same way, the amidic H atom has stronger hydrogen bonds with the carbonyl group in SCM and hence the *ν*(SN–H) frequency will be lower than in SMT-The hydrogen bonds of the amidic N–H group are stronger in SMT than in SCP (see above) and then the *ν*(SN–H) frequency will be lower in SMT than in SCP. This intermolecular interactions effect can be observed clearly comparing with frequencies calculated in isolated molecules [36], where *ν*(SN–H) appears at 3557 cm^−1^ in the molecule of SMT while appears at 3300–3293 cm^−1^ in the crystal solid form. On the contrary, this effect is smaller in SCP, where the frequency differences between isolated molecule [36] and crystal structure is smaller in *ν*(SN–H) being 3493–3439 cm^−1^, and 3431–3430 cm^−1^ in molecule and solid crystal, respectively. On the other hand, the CH atoms of the heterocycle of SCP have strong interactions with the N atoms of the heterocycle of the vicinal molecule and hence the δ(CH) vibration mode will require more energy and the frequencies will be higher than in SMT.

In SMX, again no significant differences were observed between the frequencies calculated with several *k* points and those calculated at the Γ point of the Brillouin zone of crystal lattice of polymorph III .No significant differences are observed in most of the calculated frequencies between the SMX polymorphs (Table S4). Nevertheless, the frequencies of the *ν*(NH_2_)_*as*_, *ν*(NH_2_)_*s*_, and *ν*(SN–H) normal modes of the forms I and IV are slightly higher than those of forms II and III. However, the *ν*(SN–H) frequency in the forms II and III is drastically smaller than in forms I and IV due to the intermolecular interactions of this group, especially in form II. In these vibration modes the calculated frequencies of forms II and III are closer to experimental values than those of forms I and IV. This is consistent with the higher packing energy and more stability of forms II and III than I and IV. The role of intermolecular interactions can be also detected comparing with the frequencies of the SMX isolated molecules [36], where strong differences exist in all *ν*(N–H) vibration modes, being 3644 cm^−1^ *ν*(NH_2_)_*as*_, 3531 cm^−1^ *ν*(NH_2_)_*s*_, and 3458 cm^−1^ *ν*(SN–H) in isolated SMX molecule, and 3562–3540 cm^−1^ *ν*(NH_2_)_*as*_, 3455–3374 cm^−1^ *ν*(NH_2_)_*s*_, and 3428–2917 cm^−1^ *ν*(SN–H) in solid crystal. Also, the ν(CH) frequencies of the C-H bond of the heterocyclic ring of the polymorphs I and IV are lower than in the forms II and III due to the strong intermolecular interaction with the heterocyclic N atom (see above). On the other hand, the ν(SO)_*as*_ frequencies of the polymorphs I and IV are lower than in the forms II and III, also due to the different intermolecular interactions in the crystal packing. In the same way, the ν(NO) and γ(NH) frequencies of forms I and IV are also lower than in forms II and III.

In the whole series studied the *ν*(NH_2_) frequencies follow the sequence: SMT < SCM < SMX < SCP. The only difference between these arylsulfonamides is in the substituents of SNH group, which are too far away from the amino group for possible electronic effects on this *ν*(NH_2_) vibration mode. We consider that the frequency differences should be due to variations in the intermolecular interactions of the crystal structures. This phenomenon is more clear in ν(SN–H) where the frequency sequence is: SCP > SMX-IV > SMX-I > SMT > SCM > SMX-III > SMX-II.

The complementary Raman spectra were also calculated. In general, the calculated frequencies are consistent with experimental data [44]. Some Raman spectra can be found in the Supplementary Support section (Table S2 and Fig. S5). The *ν*(NH_2_) frequencies follow the sequence: SMT < SCP < SCM < SMX-III < SMX-IV, whereas the *ν*(SN–H) frequencies follow the sequence: SCM < SMX-IV < SCP < SMX-III < SMT.

## Conclusions

Our DFT calculations reproduce the crystal structure of arylsulfonamides determined experimentally. These calculations have showed that some of the claimed crystal polymorphs are the same structure, and they are not new polymorphs. Only four different crystal forms of SMX should be considered as real polymorphs until now, being the form II the most stable. The packing energy is similar for all sulfonamides crystal studied being slightly higher in SMX, where this packing energy is higher than the energy difference between tautomeric molecular forms and the energy barrier of intramolecular tautomeric transition. Therefore, some intermolecular H atoms exchanges can be produced within some crystal structures of SMX forming tautomeric equilibria.

The main intermolecular interactions in all crystal forms of these arylsulfonamides are hydrogen bonds among the sulfonic and amino groups and SNH groups, some π-π interactions between rings and also electrostatic forces. The calculation of IR frequencies of the real crystal forms yields good agreement with experimental values and becomes a great tool for the assignment of some bands found experimentally, because the crystal models are a more realistic representation of experiment that works on solid state. In these 3-D periodical models the intermolecular interactions are included and some of these interactions can alter the vibration modes of the molecules.

## Supplementary Information

Below is the link to the electronic supplementary material.

Supplementary file1 (DOCX 212 KB)

## Data Availability

The datasets generated during and/or analysed during the current study are available from the corresponding author on reasonable request.
