# Supplementary material for: Crystal polymorphism and spectroscopical properties of sulfonamides in solid state by means of First Principles calculations
Source: J Comput Aided Mol Des. 2022 Jul 26;36(7):549–62. doi: 10.1007/s10822-022-00465-2 (PMC9399194; doi:10.1007/s10822-022-00465-2)
Supplement: Supplementary file 1 — Supplementary file1 (DOCX 212 KB) [file 10822_2022_465_MOESM1_ESM.docx]

**SUPPLEMENTARY INFORMATION**

**Crystal polymorphism and spectroscopical properties of sulfonamides in solid state by means of First Principles calculations**

Sainz-Díaz, C. Ignacio*^a^**, Pérez de la Luz, Alexander*^a,b^*, Barrientos-Salcedo, Carolina*^c^*, Francisco-Márquez, Misaela*^d^*, Soriano-Correa, Catalina *^a,e^**

*^a^* Instituto Andaluz de Ciencias de la Tierra, Consejo Superior de Investigaciones Científicas-Universidad de Granada, Av. de las Palmeras, 4, 18100-Armilla, Granada, Spain.

*^b^* Departamento de Química, Universidad Autónoma Metropolitana-Iztapalapa. Av. San Rafael Atlixco 186, Col. Vicentina, Ciudad de México, 09340, México.

*^c^* Laboratorio de Química Médica y Quimiogenómica, Universidad Veracruzana, C.P. 91700, Veracruz, Mexico.

*^d^* Instituto Politécnico Nacional-UPIICSA, Té 950, Col. Granjas México, C.P. 08400 Mexico City, Mexico.

*^e^* Unidad de Química Computacional, Facultad de Estudios Superiores Zaragoza, Universidad Nacional Autónoma de México, Iztapalapa, C.P. 09230 Mexico City, Mexico.

* [ci.sainz@csic.es](mailto:ci.sainz@csic.es); [csorico@comunidad.unam.mx](mailto:csorico@comunidad.unam.mx)

**Figure S1.** Relationship between total energy and E-cut-off (a), and rho-cutoff (b) for the crystal structure of sulfamethoxazole calculated with QE.


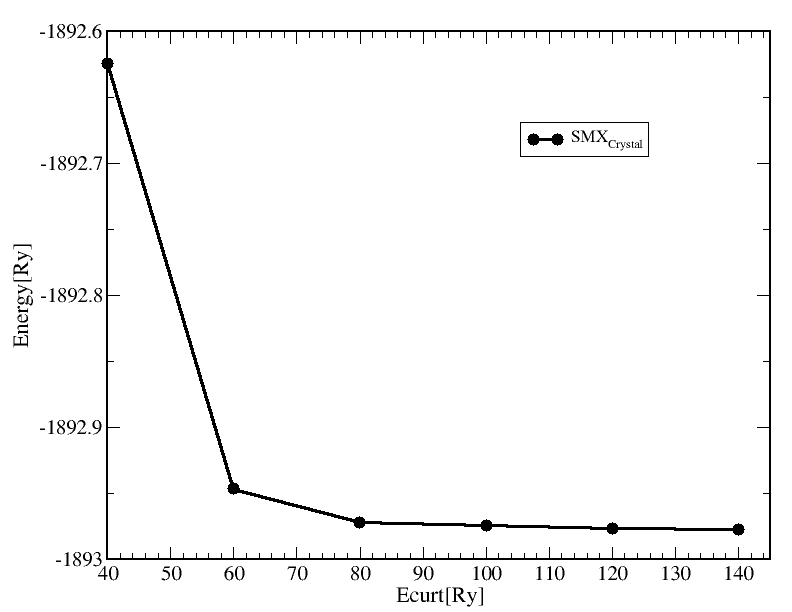
a
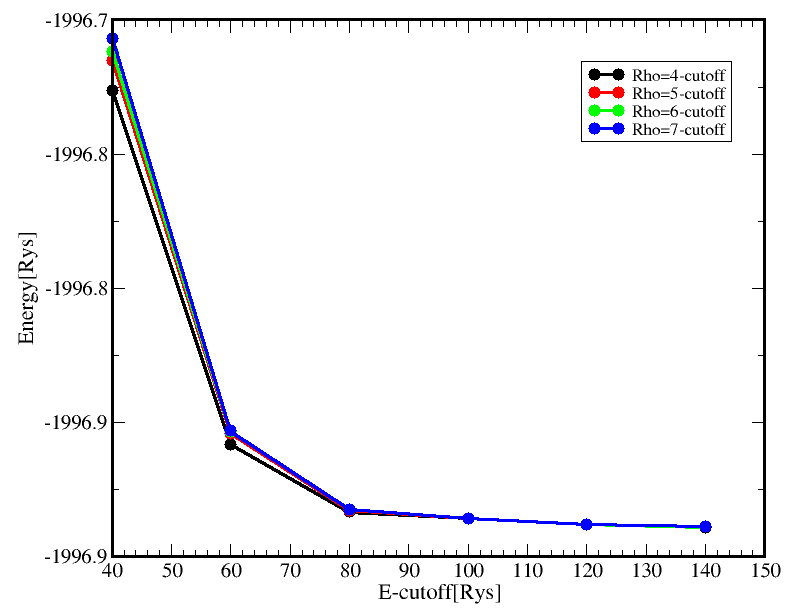
b

**Table S1.** Energy differences calculated with QE (in kcal/mol) with respect to the Γ point using several *k* points sets in the crystal orientations of some sulfonamides. The selected values are highlighted in bold style.

| Grid *k* points | Num. *k* points | SMT | SMX form I | SMX form IV | SCP |
| --- | --- | --- | --- | --- | --- |
| 111 | 1 | 0 | 0 | 0 | 0 |
| 112 | 2 | -5.299 | -0.001 | 0.020 | -0.001 |
| 113 | 2 | **-5.325** | -0.001 | 0.020 | -0.001 |
| 121 | 2 | -0.003 | -60.327 | 0.005 | -102.64 |
| 122 | 4 | -5.301 | -60.328 | 0.028 | -102.64 |
| 123 | 4 | -5.327 | -60.328 | 0.028 | -102.64 |
| 131 | 2 | -0.003 | **-61.761** | 0.006 | **-104.296** |
| 132 | 4 | -5.301 | -61.761 | 0.028 | -104.296 |
| 133 | 5 | -5.326 | -61.761 | 0.029 | -104.296 |
| 211 | 2 | 0.709 | -0.041 | -33.399 | -0.036 |
| 212 | 4 | -4.487 | -0.041 | -33.407 | -0.037 |
| 213 | 4 | -4.510 | -0.041 | -33.406 | -0.037 |
| 221 | 4 | 0.706 | -60.339 | -33.398 | -102.665 |
| 222 | 8 | -4.489 | -60.339 | -33.404 | -102.666 |
| 223 | 8 | -4.512 | -60.339 | -33.404 | -102.666 |
| 231 | 4 | 0.706 | -61.772 | -34.183 | -104.321 |
| 232 | 8 | -4.489 | -61.773 | -34.193 | -104.321 |
| 233 | 8 | -4.512 | -61.773 | -34.193 | -104.321 |
| 311 | 2 | 0.705 | -0.041 | **-34.182** | -0.036 |
| 312 | 4 | -4.488 | -0.041 | -34.190 | -0.037 |
| 313 | 5 | -4.511 | -0.041 | -34.190 | -0.036 |
| 321 | 4 | 0.703 | -60.339 | -34.181 | -102.665 |
| 322 | 8 | -4.489 | -60.339 | -34.190 | -102.665 |
| 323 | 10 | -4.513 | -60.339 | -34.189 | -102.665 |
| 331 | 4 | 0.703 | -61.773 | 0 | -104.321 |
| 332 | 8 | -4.489 | -61.773 | 0.020 | -104.321 |

**Table S2.-** Calculated Raman frequencies (in cm^-1^) of the optimised of sulfonamides (experimental values are in brackets).

| **Mode*^a^*** | **SMT** | **SCM*^b^*** | **SCP*^c^*** | **SMX-III*^d^*** | **SMX-IV*^d^*** |
| --- | --- | --- | --- | --- | --- |
| ***ν*(NH_2_)*_as_*** | 3485 | 3530 (3480) | 3493 | 3540 | 3562 |
| ***ν*(NH_2_)*_s_*** | 3301 | 3398 (3381) | 3423 | 3407 | 3440 |
| ***ν*(SN-H)** | 3281, 3269 | 3113 (3074) | 3232 (3393) | 3243 | 3196 |
| ***ν*(CH)_het_** | 3169, 3127 |  | 3160 | 3139, 3109 | 3118, 3104 |
| ***ν*(CH)_arom_** | 3082, 3047 | 3036 (3054) | 3119 (3071) | 3042, 3010 | 3038 |
| ***ν*(CH)*_as_* CH_3_** | 2984 |  |  | 2967 | 2980 |
| ***ν*(CH)*_s_* CH_3_** | 2971 | 3000 (2926) |  |  |  |
| **ν(C=O)** |  | 1660 (1688) |  |  |  |
| **δ(NH_2_)*_s_*** | 1631 | 1635 (1645) | 1657 (1629) | 1634 (1630) | 1626 (1630) |
| **Ring _arom_** | 1597 | 1597 (1598) | 1601 (1593) | 1588 (1591) | 1603 (1591) |
| **Ring _het_** | 1571 |  | 1555 (1575) | 1540 | 1543 |
| **δ(CH_)arom_** | 1533 |  | 1504 (1505) | 1505 (1508) | 1493 (1508) |
| **δ(CH)_het_+ δ(NH)** | 1497 | 1494 (1506) | 1447, 1415 (1442-1332) | 1487 | 1467 |
| **δ(CH_3_)*_s_*** | 1453 | 1460 (1506) |  | 1462 | 1424 |
| **δ(CH_3_)*_as_*** | 1417, 1402 | 1405 |  | 1437, 1415 |  |
| **ν(CN)** | 1362, 1323 | 1339 (1325) | 1377, 1353 (1388-1308) | 1377, 1334 | 1364, 1343 |
| **δ(CH)_het_** | 1126 |  | 1323 (1332) | 1126 | 1127,1293 |
| **ν(SO)*_as_*** | 1256 | 1271 (1193) | 1272 | 1273 | 1271 |
| **ν(C-O)** |  |  |  | 1248 | 1253 |
| **δ(CH)_arom_** | 1294, 1167 | 1177 (1151) | 1171 (1189) | 1171 | 1183 |
| **ν(C-Cl)** |  |  | 1126 |  |  |
| **ν(SC) + ν(SO)*_s_*** | 1097, 1072 | 1101 (1093) | 1103, 1073 (1086) | 1099 (1141) | 1096, |
| **δ(NH_2_)*_as_*** | 1051 | 1060 | 1057 (1037) | 1059 | 1056 |
| **γ(CH_3_)** | 1020 | 1043 |  | 1025 | 1029 |
| **γ(CH)_het_** | 986 |  | 1015, 991, 944 | 994 | 1006, 990 |
| **ν(NO)** |  |  |  | 916 | 946 |
| **γ(CH)_arom_** | 964 | 976 | 921, 882 (906) | 873 | 895 |
| **ν(NS)** | 948 | 858 (837) | 835 | 832 | 848 |
| **γ(NH)** | 834 | 822 | 816 | 816 | 817, 765 |

*^a^* Normal vibration modes, *as* = antisymmetric, *s* = symmetric, arom = aromatic, het = heterocyclic. *^b^* Experimental data in brackets from Goud et al. [43] *^c^* Experimental data in brackets from Basha et al. [42] *^d^* Experimental data in brackets from Lai et al. [44]

**Figure S2.** Powder X-ray diffractograms of SMT simulated from the experimental and optimized crystal structures.


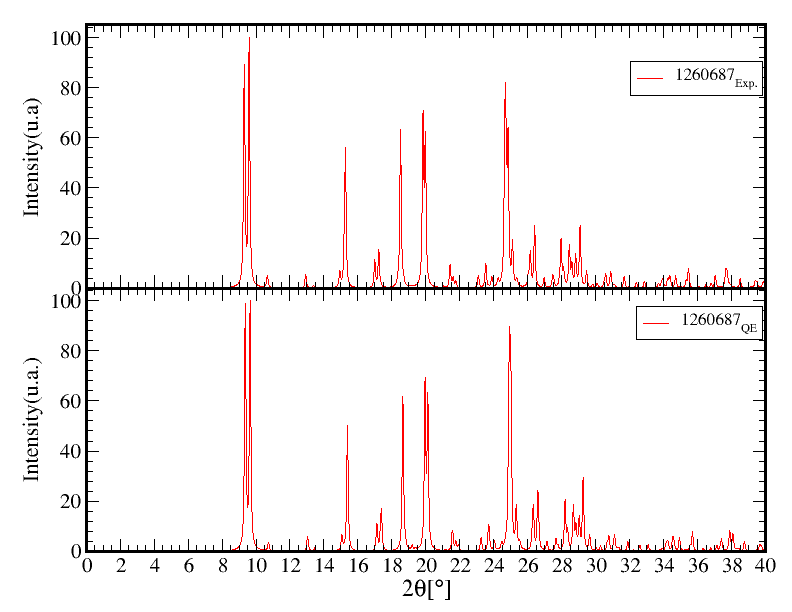


**Figure S3.** Powder X-ray diffractograms of the SMX polymorphs simulated from the experimental and optimized crystal structures: SMX form I (a), SMX form II (b), SMX form III (c), and SMX form IV (d).


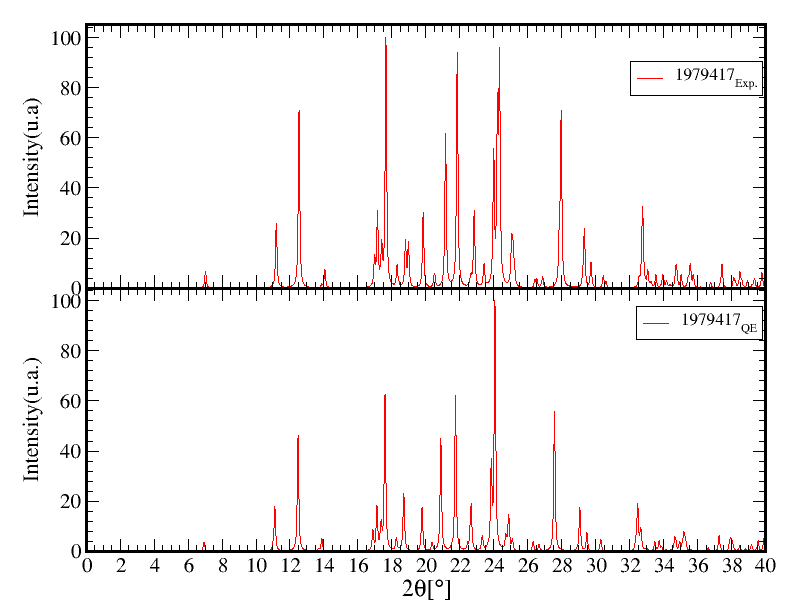
a


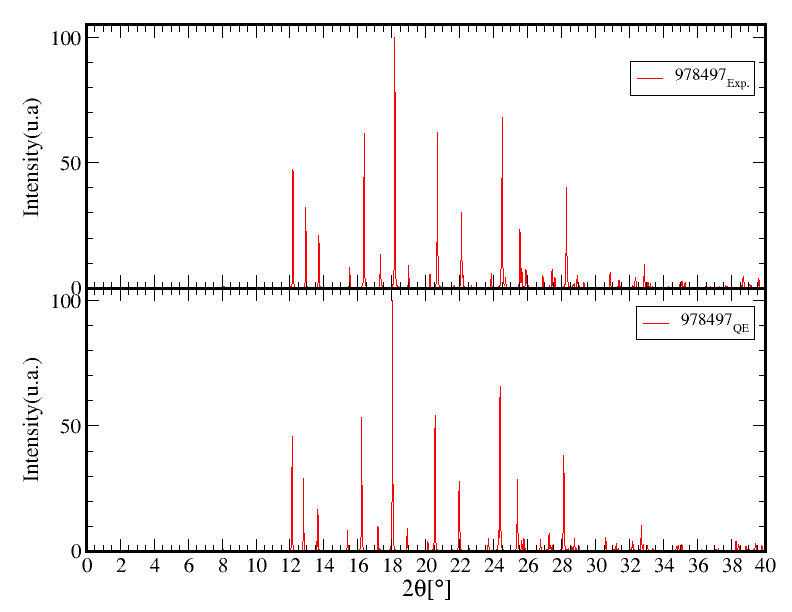
b


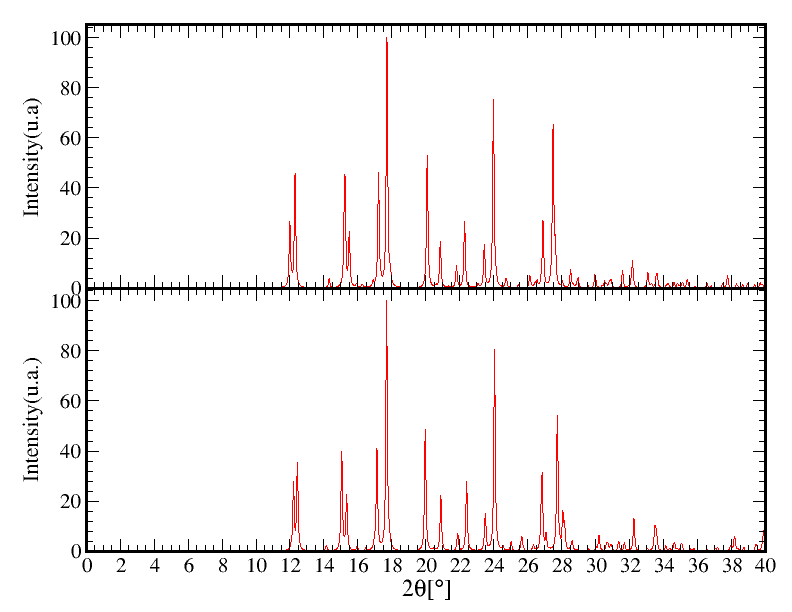
c


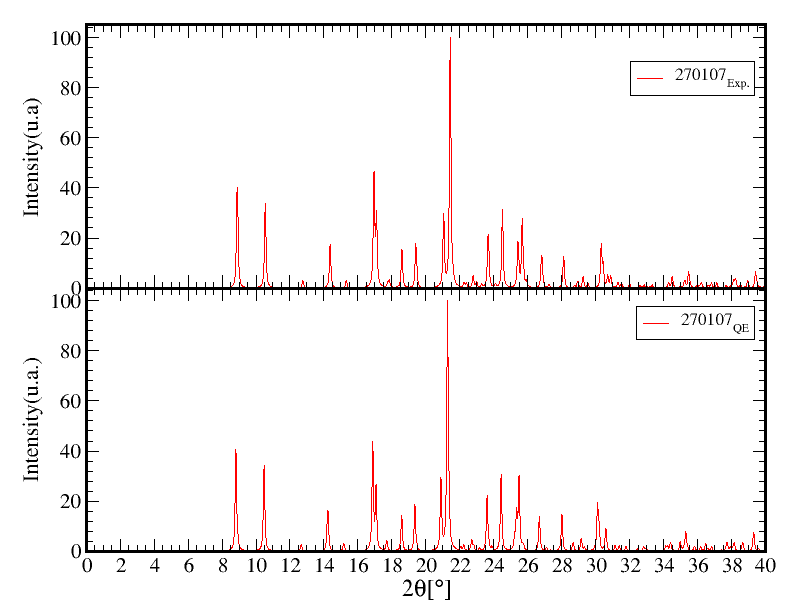
d

**Figure S4.** Powder X-ray diffractograms of the SCP polymorphs and SCM simulated from the experimental and optimized crystal structures: SCP form I (a), SCP form II (b), and SCM (c).


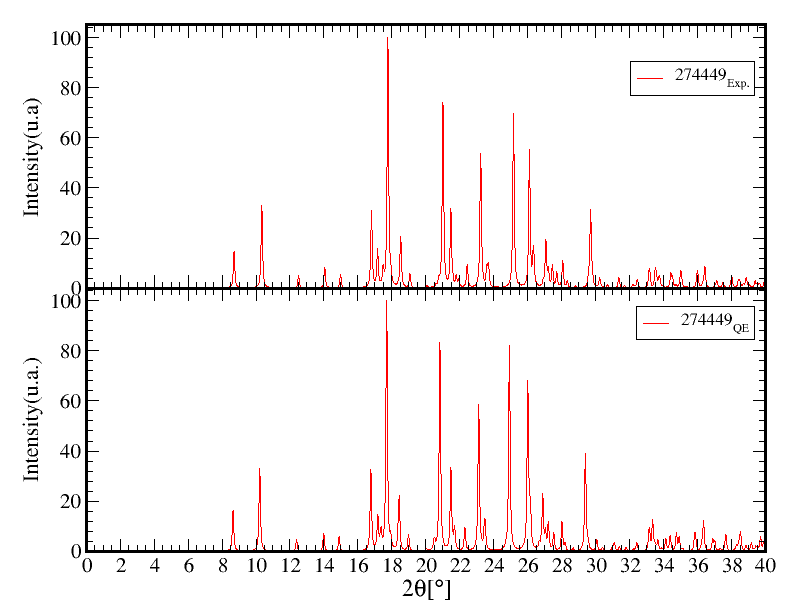
a


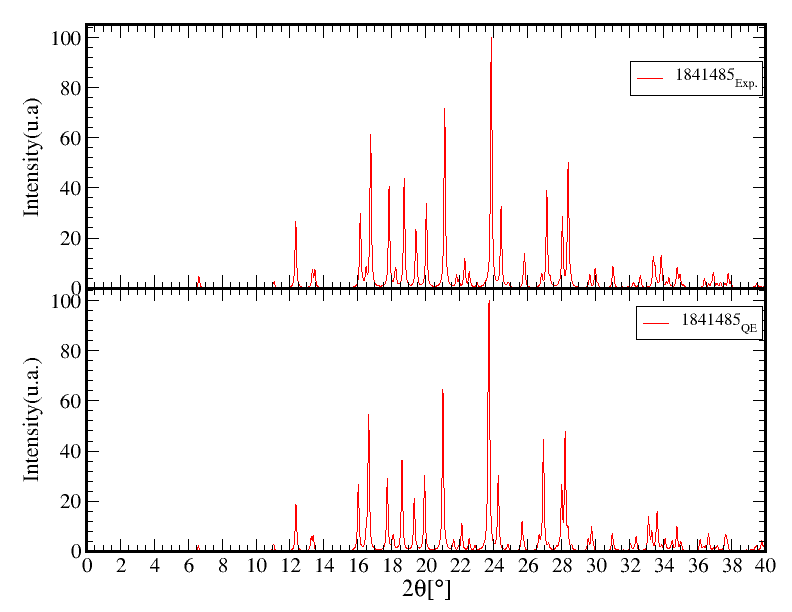
b


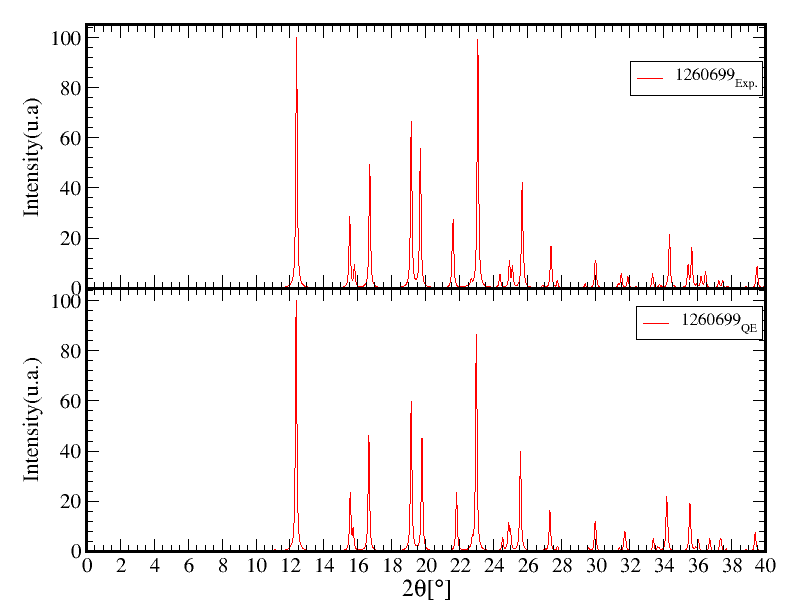
c

**Table S3.**- Calculated and experimental IR frequencies (cm^-1^) of SMT, SCM, and SCP.

| **Mode *^a^*** | **SMT_exp_*^b,c,d^*** | **SMT*^e^*** | **SCM_exp_*^m^*** | **SCM*^e^*** | **SCP_exp_*^n^*** | **SCP-I*^e^*** |
| --- | --- | --- | --- | --- | --- | --- |
| ***ν*(NH_2_)*_as_*** | 3423, 3442*^c^*-3460*^d^* | 3498-3496 | 3471 | 3532-3528 | 3494, 3496*^o^* | 3598-3596 |
| ***ν*(NH_2_)*_s_*** | 3355, 3342*^c^* | 3315-3304 | 3381 | 3405-3397 | 3392, 3316*^o^* | 3485-3480 |
| ***ν*(SN-H)** | 3259, 3240*^c^*-3260*^d^* | 3294*^p^*, 3282 | 3114-3040 | 3051, 3036-3000 | 3317, 3394*^o^* | 3431-3430 |
| ***ν*(CH)_het_** | 3075, 3085*^d^* | 3170-3169 |  |  | 3075*^o^* | 3161-3160*_s_*, 3153-3152*_as_* |
| ***ν*(CH)_arom_** | 3102, 3100*^d,^* 3066-3039, 3060-3030*^d^* | 3099*^i,j^*, 3130*^g,j^*, 3127*_s_ ^h^*, 3117*_as_^h^* | 3160 | 3142-3115, 3112 | 3137*^o^* | 3141*_s_*, 3133-3114 |
| ***ν*(CH)*_as_* CH_3_** | 2920*^d^* | 3095-3032*^j^*, 3079*^h^* | 3114 | 3113, 3046 |  |  |
| ***ν*(CH)*_s_* CH_3_** | 2900*^d^* | 2991*^j^*, 2967*^h^* | 2918 | 2982 |  |  |
| **ν(C=O)** |  |  | 1686 | 1669-1636 |  |  |
| **δ(NH_2_)*_s_*** | 1652, 1605*^d^* | 1633-1628 | 1640 | 1634-1624 | 1625, 1626*^o^* | 1624-1595 |
| **Ring _arom_** | 1594, 1580, 1615*^d^* | 1601-1593*_s_*, 1545, 1438, 1354 | 1596, 1505, 1419 | 1599-1595*_s_*, 1549,1443, 1363, 1494 | 1595, 1596*^o^* | 1624-1595, 1544, 1501-1450, 1366-1364, 988 |
| **Ring _het_** | 1585, 1660*^d^* | 1581-1569, 1333-1329 |  |  | 1574, 1574*^o^*, 1505*^o^*, 1330 | 1572-1551, 1376, 1230, 1157, 1029 |
| **δ(CH)_arom_** | 1501, 1492, 1560*^d^* | 1438, 1498 | 1155 | 1292, 1180-1176*_s_*, 1130-1117*_as_* | 1506, 1439*^o^* | 1293, 1181-1179, 1126-1124 |
| **δ(CH)_het_+ δ(NH)** | 1408, 1510*^d^* | 1454-1451, 1325 | 1469 | 1465-1455*^f^*  1247-1244*^f^* | 1435, 1392*^o^* | 1551, 1422-1417, 1378, 1342, 1228 |
| **δ(CH_3_)*_s_*** | 1480*^d^*, 1330-1300*^d^* | 1365-1327 | 1322 | 1351-1350 |  |  |
| **δ(CH_3_)*_as_*** | 1420*^d^* | 1440, 1432-1415 | 1375 | 1465-1403 |  |  |
| **ν(CN)** | 1262,1304*^d^*, 1390*^d^* | 1533-1526*_s_*, 1497*^k^*, 1403-1393*^f^*, 1344-1338*^k^*, 1075-1072*^f^*, 1257-1242 | 1245 | 1341-1338*^k^*, 1271-1268, 1247-1244 | 1309*^o^* | 1500*^k^*, 1348-1346*^k^*, 1338 |
| **δ(CH)_het_** | 1093, 1190*^d^* | 1171 |  |  |  | 1422-1417, 1342-1338 |
| **δ(CH)_arom_** | 1187, 1160-1130*^d^* | 1295, 1179-1177, 1127 | 1191, 1155 | 1292, 1180-1176*_s_*, 1130-1117 | 1190*^o^*, 1144*^o^* |  |
| **ν(SO)*_as_*** | 1326, 1300*^d^* | 1257-1211 | 1245 | 1271-1268 | 1350, 1240*^o^* | 1274-1270 |
| **ν(N-N)** |  |  |  |  |  | 1204-1202 |
| **ν(C-Cl)** |  |  |  |  |  | 1155, 1092-1088 |
| **ν(SC) + ν(SO)*_s_*** | 1157, 1100*^d^* | 1102-1096, 1057-1052 | 1091 | 1111-1057 | 1330, 1086*^o^* | 1122-1098, 1059-1055 |
| **δ(NH_2_)*_as_*** | 1080*^d^* | 1034 | 1040 | 1044 |  | 1031 |
| **γ(CH_3_)** | 980*^d^* | 1023-1009 | 995 | 1023, 976 |  |  |
| **γ(CH)_het_** | 971, 880*^d^* | 845-842 |  |  | 906*^o^*, 825*^o^* | 1018-1015, 880-873 |
| **γ(CH)_arom_** | 971, 880*^d^* | 961-950 |  | 950-932 | 966*^o^* | 976-945, 839-819 |
| **γ(NH)** |  |  | 857 | 862-856 |  |  |
| **ν(C-CH_3_)** |  | 967-966 |  | 961-957 |  |  |
| **ν(SN)** |  |  |  |  |  | 887-883, 803-798 |

*^a^* Normal vibration modes, *as*=antisymmetric, *s*= symmetric, arom=aromatic, het=heterocyclic, umb=umbrella. *^b^* Experimental data in solid state with KBr of sulfadiazine, a similar structure without methyl groups [41]. *^c^* Values partially assigned experimentally [26]. *^d^* Values extracted from the experimental spectrum of SMT in solid state with KBr [26] without assignment reported. *^e^* Calculated at the Γ point of the Brillouin zone of crystal lattice. *^f^* S-(NH)-C group.  *^g^* In alpha position with respect to the SO group. *^h^* In the anti zone with respect to the N-H bond. *^i^* In alpha position with respect to the amino group. *^j^* In the zone syn with respect to the N-H bond. *^k^* CNH_2_. *^m^* Experimental values extracted from the spectrum of SCM [43]. *^n^* Experimental values from [14]. *^o^* From [42]. *^p^* Coupled with *ν*(NH_2_)*_s_*.

**Table S4.**- Calculated and experimental IR frequencies (cm^-1^) of SMX crystal polymorphs.

| **Mode *^a^*** | **SMX_exp_*^b^*** | **SMX-I** | **SMX-II** | **SMX-III** *^c^* | **SMX-IV** |
| --- | --- | --- | --- | --- | --- |
| ***ν*(NH_2_)*_as_*** | 3462 | 3558-3555 | 3544-3541 | 3542-3540 | 3562-3561 |
| ***ν*(NH_2_)*_s_*** | 3376 | 3455-3449 | 3428-3374 | 3427-3412, 3407-3405 | 3446-3439 |
| ***ν*(SN-H)** | 3292 | 3405-3404 | 2963-2917 | 3013-3006, 2991-2967 | 3429-3428 |
| ***ν*(CH)arom** |  | 3144-3119, 3110-3109 | 3157-3104, 3076-3071 | 3140*^d,e^*, 3118, 3109*^e,f^*, 3100*^g^* | 3133-3104 |
| ***ν*(CH)het** |  | 3195-3194 | 3246-3208 | 3243 | 3196 |
| ***ν*(CH)*_as_* CH_3_** |  | 3089-3045 | 3088-3076, 3051-3040, | 3097-3041 . | 3092-3038 |
| ***ν*(CH)*_s_* CH_3_** |  | 2985 | 2963, 2952, 2919 | 2968 | 2980 |
| **Ring het** | 1640 | 1605-1603 | 1609-1600 | 1604-1600 | 1604-1597, 1006 |
| **Ring arom** | 1610 | 1599-1550, 1373-1371, 996-995 | 1617-1542, 1350-1334 | 1598-1540, 1356 | 1606*_s_*, 1604-1542, 1450, 1365-1361, 990 |
| **δ(NH_2_)*_s_*** | 1500 | 1624-1609, 1604-1603 | 1643-1625 | 1639-1630 | 1636-1626 |
| **δ(CH)arom** |  | 1499-1494 | 1496-1482, 1462-1442 | 1492-1487, 1437-1435 |  |
| **δ(CH)het+ δ(NH)** |  | 1503-1501, 1266-1263 | 1598-1592 | 1385 | 1342-1339, 1253 |
| **δ(NH)** |  | 1343-1339 | 1514-1500, 1395-1380, 1131 |  |  |
| **δ(CH_3_)*_s_*** |  | 1370-1366 | 1380-1360 | 1388, 1360 | 1369-1361 |
| **δ(CH_3_)*_as_*** |  | 1436-1419 | 1431-1402 | 1423-1410 | 1430-1421 |
| **ν(CN)** |  | 1467-1459, 1327-1322 | 1463, 1320*^h^* | 1465, 1334*^h^* | 1502*^h^*, 1348*^h^*, 1469-1465*^i^*, 1494-1491*^j^* |
| **δ(CH)het** |  | 1186-1184 | 1182, 1151-1140 | 1125-1115 | 1186 |
| **δ(CH)arom** |  | 1298-1297, 1180-1177, 1133-1126 | 1331-1273, 1172-1041 | 1295, 1171*_s_*, 1134 | 1348, 1293, 1182*_s_*, 1127 |
| **ν(SO)*_as_*** | 1317 | 1254-1251 | 1272-1266 | 1275-1272 | 1254 |
| **ν(C-O)** |  | 1266, 973-972 | 1250-1238 | 1251-1248 | 1272, 977-975 |
| **ν(SC) + ν(SO)*_s_*** | 1317 | 1121-1100, 1063-1059 | 1117-1103, 1096-1092 | 1107-1096, 1057 | 1119-1055 |
| **δ(NH_2_)*_as_*** |  | 1133-1126, 1077-1074 | 1131 | 1129, 1052-1048 | 1047 |
| **γ(CH_3_)** |  | 1036-1006, 900-896 | 1040-1027 | 1027-1023 | 1031-1028 |
| **γ(CH)het** | 929, 828 | 909-908 | 1182-1041 | 998-995, 777 | 893-891 |
| **γ(CH)arom** |  | 986-983, 954, 847-843, 840-823 | 996-930 | 955-932 | 976-946, 833-816 |
| **ν(NS)** | 1157 | 847-843 | 847-842 | 870-867 | 851-846 |
| **ν(NO)** |  | 900-896 | 964 | 986-981, 919-916 | 899 |
| **γ(NH)** |  | 788-783 | 930,902-886 | 875-865 | 765 |

*^a^* Normal vibration modes, *as* = antisymmetric, *s* = symmetric, arom = aromatic, het = heterocyclic. *^b^* Experimental data from [41]. *^c^* All values of the table are calculated at the Γ point of the Brillouin zone of crystal lattice.  *^d^* In alpha position with respect to the SO group. *^e^* In the zone anti with respect to the N-H bond. *^f^* In alpha position with respect to the amino group. *^g^* In the zone syn with respect to the N-H bond. *^h^* CNH_2_. *^i^* SC-NH group. *^j^* Heterocyclic C-N bonds.

**Figure S5.** Raman spectra calculated for SMT (a), SMX-III (b) SMX-IV (c), and SCP (d).


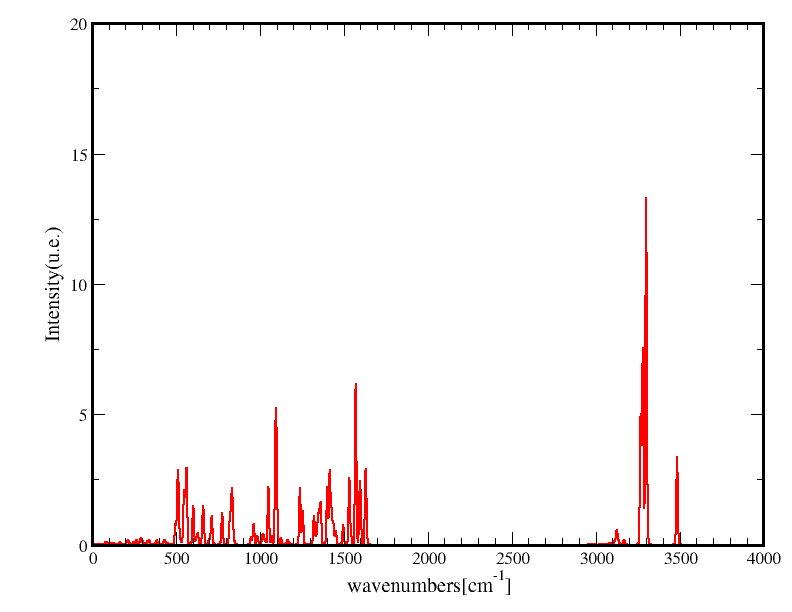
a


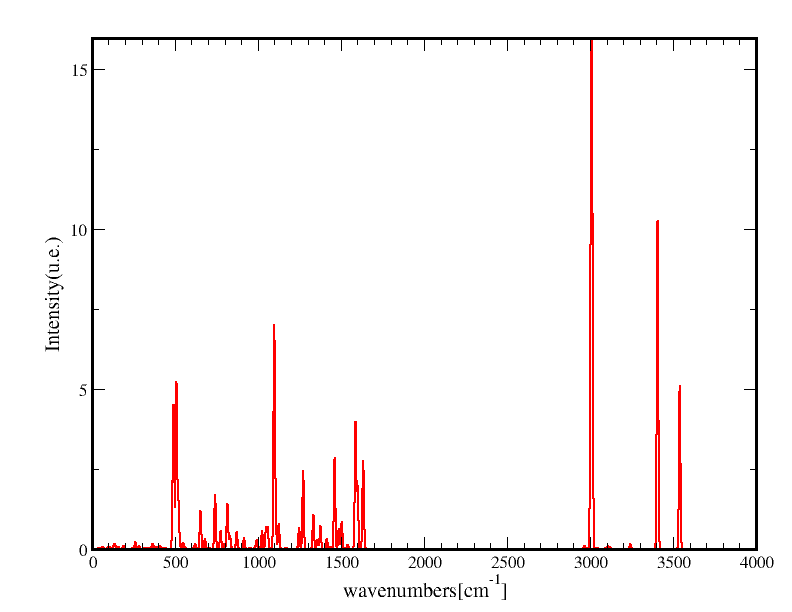
b


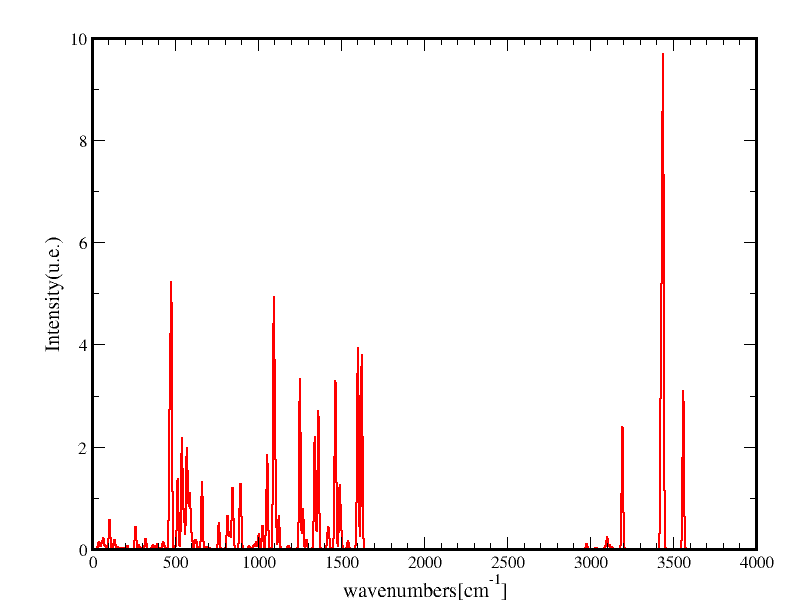
c


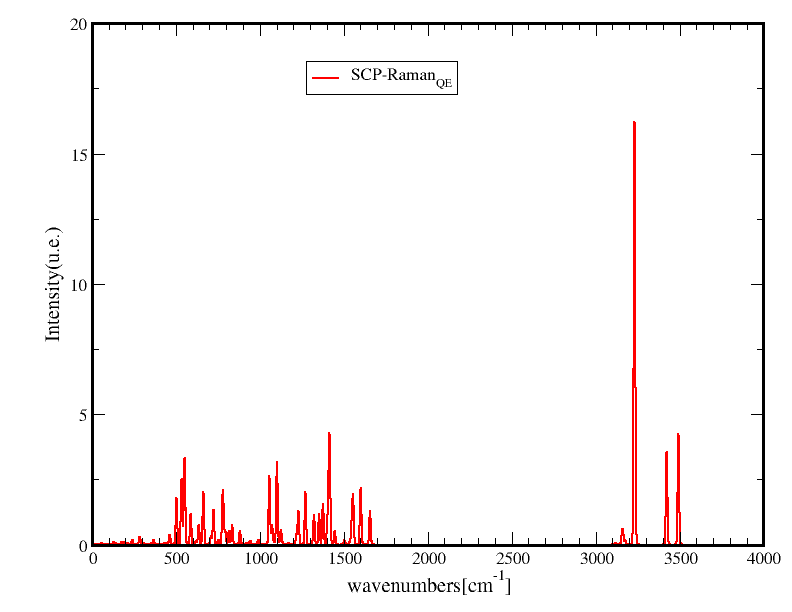
d
